# Gastrodin From *Gastrodia elata* Enhances Cognitive Function and Neuroprotection of AD Mice *via* the Regulation of Gut Microbiota Composition and Inhibition of Neuron Inflammation

**DOI:** 10.3389/fphar.2022.814271

**Published:** 2022-06-02

**Authors:** Opeyemi B. Fasina, Jianyu Wang, Jianxia Mo, Hiroyuki Osada, Hiroshi Ohno, Wensheng Pan, Lan Xiang, Jianhua Qi

**Affiliations:** ^1^ College of Pharmaceutical Science, Zhejiang University, Hangzhou, China; ^2^ Chemical Biology Research Group, RIKEN Center for Sustainable Resource Science, Wako, Japan; ^3^ Laboratory for Intestinal Ecosystem, RIKEN Center for Intestinal Ecosystem, Yokohama, Japan; ^4^ Department of Gastroenterology, Zhejiang Provincial People’s Hospital, People’s Hospital of Hangzhou Medical College, Hangzhou, China

**Keywords:** AD mice, gut microbiota, memory, neuroinflammation, microglial cell

## Abstract

Gastrodin (Gas) is known to exhibit neuroprotective effects in Alzheimer’s disease (AD). However, the detailed mechanism of action is still unclear. In the present study, we focused on the microbiome–gut–brain axis to investigate the mechanism of action of Gas using a D-galactose (Dgal)–induced AD model. Gas reversed the memory dysfunction of Dgal-administered mice. Neurons in the cerebral cortex and hippocampus were reduced in the Dgal-administered group, and the decrease of neurons was suppressed in 90 and 210 mg/kg Gas treatment groups. 16S rRNA sequence analysis was carried out to explore the composition of gut microbiota in fecal samples of mice. Gas treatment had a positive correlation with Firmicutes and had a negative correlation with Cyanobacteria, Proteobacteria, and Deferribaceters*.* Importantly, the LPS and proinflammatory cytokines in the brain increased in Dgal-administered mice, but these parameters recovered to normal levels after oral administration of Gas. To determine whether the microbiota–gut–brain axis is involved in the neuroprotective effect of Gas, the mice were given antibiotic cocktail before and during the trial period to decrease the gut microbiota of mice. The antibiotic cocktail partially eliminated the neuroprotective effect of Gas by changing the gut microbiome composition. These results indicated that Gas improves the memory of the AD mouse model *via* partly targeting the microbiota–gut–brain axis and mitigating neuron inflammation.

## Introduction

The elderly population is growing at an unprecedented rate. Currently, there are about 703 million people above 65 years of age worldwide, which is estimated to progressively increase to more than two billion by 2050 (United Nations, 2019). Aging is a complicated progressive debilitation of physiological functions, which induces the degeneration of organ functions and exhibits several phenotypes that promote diseases such as cancer, type 2 diabetes, and hypertension (Xiao et al., 2018; Zhang et al., 2018). Alzheimer’s disease (AD) is an important manifestation of aging, especially pathological brain aging. It is a pathological syndrome that is primarily characterized by major symptoms such as impaired cognitive function and perturbed daily life performance with behavioral abnormalities (Akhtar and Sah, 2020). The current drugs for AD, including four cholinesterase inhibitors and one N-methyl-D-aspartate (NMDA) receptor antagonist, have been able to mitigate AD symptoms but cannot cure the disease (Iraji et al., 2020). Thus, new therapeutic targets and drugs for the treatment and prevention of AD should be identified.

Animal models play an important role in studying AD. To date, transgenic mice such as APP/PS1, 3xTg-AD mice (Virgili et al., 2018), scopolamine- and D-galactose (Dgal)–induced mice (Li et al., 2016; Ali et al., 2020) are used to perform efficacy evaluation and determine the mechanism of action of certain compounds. Dgal, a normal reducing sugar, is widely used to induce age-related diseases, including AD (Oskouei et al., 2021). Rodents administered with Dgal show cognitive dysfunction, increased free radical production, mitochondrial dysfunction, and impaired calcium homeostasis (Jeong et al., 2021). Recently, some studies reported that Dgal promotes inflammation, neurodegeneration, and gut microbiota dysbiosis (Xiao et al., 2018; Zhang et al., 2018; Ali et al., 2020). In the present study, we used Dgal-induced AD mice for our experiments.

With the understanding of gut microbiota, more and more pieces of evidence indicate that the gut–brain axis plays a vital role in neuronal development and neurodegenerative diseases (Kennedy et al., 2017; Zhang et al., 2018). The status of gut function and the composition of gut microbiota can influence brain function and diseases. Several studies have reported the impact of dysbiosis on several neurodegenerative diseases such as AD and Parkinson’s disease (Kennedy et al., 2017; Liu et al., 2020). Gut microbiota dysbiosis has been hypothesized to promote proinflammatory cytokines, induce neurotoxins, and cause metabolic disturbances (Tan et al., 2021). On the contrary, gut microbes, such as Bifidobacteria, Firmicutes, Actinobacteria, and Verrucomicrobia, have been implicated in promoting cognitive function (Sharma et al., 2021). Furthermore, several studies have shown the marked difference in gut microbiota composition and diversity in AD patients compared with those in their respective control (Sharma et al., 2021). The variance in the gut microbiota between diseased and healthy individuals in several diseases makes it a promising target for prevention and treatment of diseases. In addition, the promotion of dysbiosis leads to increased pathogenic bacteria that induce brain damage, while eubiosis promotes the good functionality of the brain (Kennedy et al., 2017; Sharma et al., 2021).

*Gastrodia elata*, commonly known as Tian ma in Chinese, is an orchid used in traditional Chinese medicine to treat spasms, paralysis, dizziness, stroke, and dementia (Liu et al., 2018). *G. elata* contains gastrodin (Gas), parishin, p-hydroxybenzyl alcohol, vanillin, and vanillyl alcohol compounds, but the principal bioactive compound is Gas. Since it was first isolated in 1978, extensive investigations on the biological activity of Gas have been performed (Liu et al., 2018). Gas possesses a broad range of beneficial effects on neurodegenerative diseases, and its mechanisms of actions include modulating neurotransmitters, exerting antioxidative and anti-inflammatory effects, suppressing microglial activation, regulating mitochondrial cascades, and upregulating neurotrophins (Liu et al., 2018). Although the brain–gut–microbiota axis is an emerging pharmacological target in AD, the effect of Gas on the gut–brain axis remains unknown. In this study, we show that Gas treatment can promote cognition and neuroprotection and mitigates neuron inflammation by regulating the gut microbiota composition of AD model mice. To our knowledge, this study is the first to assess the influence of Gas on cognition by regulating gut microbiota.

## Materials and Methods

### Drugs and Reagents

Dgal and donepezil (Done) were purchased from Sangon Biotech Co., Ltd., Shanghai, China and Sigma-Aldrich Co., St. Louis, MO, United States, respectively. Antibiotics (vancomycin, neomycin, ampicillin, and metronidazole) and 4′,6-diamidino-2-phenylindole (DAPI) were obtained from Macklin Shanghai, China. Tumor necrosis factor alpha (TNF-α), interleukin-1 beta (IL-1β), IL-6, lipopolysaccharide (LPS), and ELISA kits were provided by 4A Biotech, Beijing, China and Hefei Laier Biotech, Anhui, China. The antibodies of TLR4 (4A Biotech, Beijing China), occludin, pIKBα, zonulin (Santa Cruz Biotechnology, Dallas, Texas, United States), neuronal nuclei (NeuN), IKBα, Iba-1 (Cell Signaling Technology, Boston, United States), GADPH, secondary antibody (horseradish peroxidase [HRP]–linked anti-rabbit and anti-mouse IgGs), and ECL Western blot chemiluminescence detection kit (Beijing CoWin Biotech Company, Beijing, China) were used in the present study. All primers in this work were synthesized by Sangon Biotech Co., Ltd., Shanghai, China.

### Extraction and Isolation of Gastrodin

**FIGURE 1:**
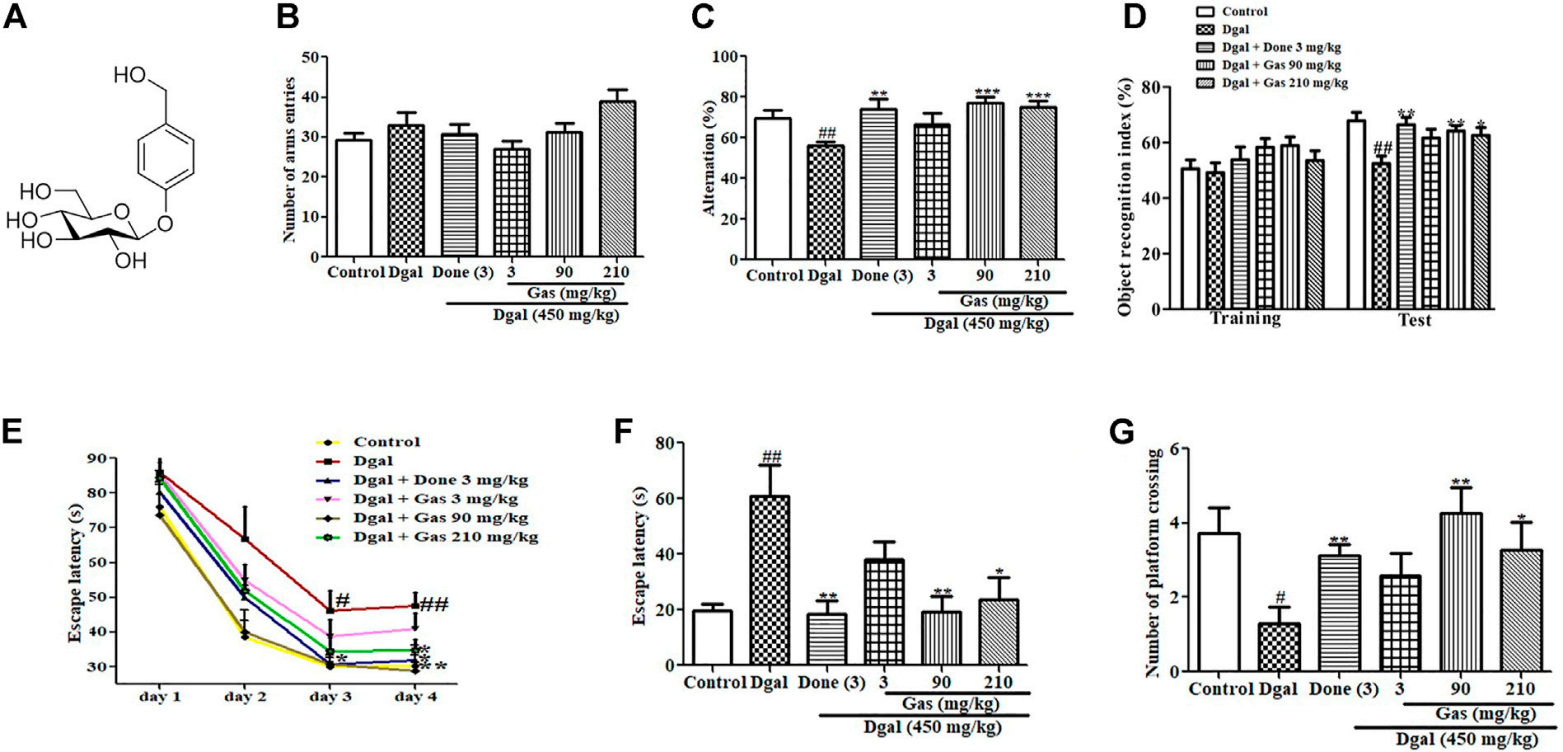
Chemical structure of Gas and changes in learning memory and spatial memory of AD mice after administering Gas. **(A)** Chemical structure of Gas. **(B)** Changes in the number of arm entries in the Y-maze test. **(C)** Alternation in the Y-maze test. **(D)** Object recognition index in the NOR experiment. **(E)** Escape latency in the training phase in the water maze test. **(F,G)** Escape latency and number of platform crossings in the water maze test. The number of animals in each group is eight, and the data are presented as means ± SEM. ^# and ##^ indicate significant differences at *p* < 0.05 and *p* < 0.01 compared with those of the normal control, respectively; ^*, **,and ***^indicate significant differences at *p* < 0.05, *p* < 0.01, and *p* < 0.01 compared with those of the Dgal group, respectively.

The dried rhizome of *Gastrodia elata* was purchased from Chengdu, Sichuan Province, China, identified by associate professor Jianxia Mo and assigned a voucher specimen number (no. 20191114) of the plant deposited at the Institute of Materia Medica, Zhejiang University. Dried *G. elata* (1.5 kg dry weight) was crushed and extracted with 100% methanol for 48 h of shaking, filtration, and concentration with a vacuum pump to obtain 135 g crude extract. Under the guidance of the bioassay system (lifespan assay of K6001 yeast) (Lin et al., 2019), the crude extract (135 g) was separated by a silica gel (200–300 mesh, Yantai Research Institute of Chemical Industry, Yantai, China) open column and eluted with CH_2_Cl_2_/MeOH (100:0, 95:5, 90:10, 85:15, 80:20, 75:25, 70:30, 60:40, and 0:100). The active fraction (12 g) eluted with CH_2_Cl_2_/MeOH (75:25) was purified by HPLC (H&E SP ODS-A (Φ = 20 × 250 mm)) with 5% of aqueous methanol at a flow rate of 8 ml/min to obtain an active compound (*t*_R_ = 41 min) in batches. The chemical structure of the active compound was identified to be Gas by analyzing HR ESI-MS and ^1^H NMR spectra and compared with that of the reported one (Lee et al., 2015). The chemical structure is given in Figure 1A.

### Animal and Experimental Design

Gas and Done were dissolved in water prior to oral administration, while Dgal was dissolved in normal saline prior to intraperitoneal administration. Sixty Institute of Cancer Research (ICR) mice aged 8 weeks’ old were purchased from Zhejiang Academy of Medical Sciences, Hangzhou, China. They were acclimatized under standard condition of 23°C temperature, 50% humidity, and 12 h light/dark cycle and provided chow diet and water *ad libitum*. After 1 week of acclimatization, the mice were randomly divided into the following groups: normal control, Dgal group, Dgal + Done 3 mg/kg group, Dgal + Gas 3 mg/kg, Dgal + Gas 90 mg/kg, and Dgal + Gas 210 mg/kg groups. The control and Dgal groups were given water, while others were given the respective drug dosage *via* oral gavage daily for 9 weeks. In addition, the control group was given normal saline, and all other groups were given Dgal dissolved in normal saline at 450 mg/kg *via* intraperitoneal injection daily for 9 weeks. The Y-maze and novel object recognition (NOR) tests started at the eighth week, while the Morris water maze (MWM) test was started at the ninth week. The mice were killed at the end of the ninth week. Blood was taken *via* the orbital sinus, and brains and intestines of some mice were removed and preserved in a −30°C refrigerator for further analysis. At the same time, three mice in each group were perfused with cold PBS followed by cold 4% paraformaldehyde (PFA). Thereafter, the brains were embedded and sectioned for immunohistochemical staining.

In addition, the second study was carried out using 8-week-old male ICR mice. Forty mice were divided into the following groups: normal control, Dgal group, Dgal + Gas 90 mg/kg group, and Dgal + Gas 90 mg/kg + ABX group. The ABX group was administered with 100 µl of antibiotic (ABX) cocktail, which includes 0.5 g/L of vancomycin, 1 g/L of neomycin, 1 g/L of ampicillin, and 1 g/L of metronidazole, for 2 weeks in order to deplete the gut microbiota. The other groups were administered with 100 µl of Milli-Q water for 2 weeks. After 2 weeks, the normal control and Dgal group were given Milli-Q water *via* oral gavage, while other groups were administered with 90 mg/kg of Gas daily for 9 weeks. However, the Dgal + Gas 90 mg/kg + ABX group was treated with 100 µl of ABX cocktail daily for 9 weeks. The normal control group was treated with normal saline, and all other groups were given 450 mg/kg of Dgal dissolved in normal saline *via* intraperitoneal injection daily for 9 weeks. The Y-maze and NOR tests were started at the tenth week, while the MWM test was started at the twelfth week. At the end of the animal experiment, the samples and brain were taken as mentioned previously. All animal experiments were conducted with strict accordance to the Guide for the Care and Use of Laboratory Animals of the National Institute of Health. In addition, the Committee on the Ethics of Animal Experiments of Zhejiang University Permit approved the experiment with permit number (ZJU20210155).

### Y-Maze Test

The Y-maze test is used to measure working memory, and the method of assessment has been reported in a previous study (Li et al., 2016). The Y-maze comprises three arms (A, B, and C) with 30 cm length, 10 cm width, and 15 cm height with 120^o^ relative to adjacent arms. The mouse activity in the Y-maze was recorded with a video recorder, and data were stored in ANY-MAZE software 6.35 (Stoelting, Illinois, United States). Each mouse was placed facing the wall of arm A and allowed to move freely in the maze for 5 min. The mice whose whole or half body entered an arm was recorded as an arm entry. Furthermore, an alternation was considered when a mouse moved using the three arms without revisiting the first arm. The total number of arm entries and alternations was calculated with ANY-MAZE software. The alternation was calculated by dividing the total number of alternations by the total number of arm entries −2 × 100.

### Novel Object Recognition Test

The NOR test was carried out as previously described (Li et al., 2016). Briefly, the NOR test was performed in an open-field environment with an arena of a black plastic box (50 cm × 50 cm × 50 cm) with two identical objects and a novel object. The activity of mice in the open arena was recorded with a video recorder and stored in ANY-MAZE software. During the habituation phase, each mouse was placed in the open arena without objects for 5 min. In the middle of training, the open arena was cleaned with 75% ethanol and dried. After 24 h, the NOR test was performed, which included the training and test phases. In the training phase, each mouse was placed in the same arena with two identical objects (5 cm × 5 cm × 5 cm cubic steel placed on each other) set apart at equal distance for 5 min. After 1 h, one of the identical objects was substituted with a novel object (5 cm × 5 cm cylindrical steel placed on each other), and the exploration time of each mouse was recorded. The exploration was determined by the time spent by the mice either touching or sniffing the objects. The exploration time during the training and test phases was recorded with a video recorder, and data were stored in ANY-MAZE software. The alternation was calculated by dividing time spent on novel object by total time spent on both objects × 100 was calculated by ANY-MAZE software.

### Morris Water Maze Test

The MWM test is used to measure spatial and long-term memory. It comprises a circular tank (1.25 m diameter and 50 cm depth) containing 25 cm deep warm water (22°C), a platform (10 cm diameter and submerged 1 cm below the water), a video camera recorder, and a computer. The experiment was carried out in 5 days. The mice were subjected to a 4-day acquisition experiment, where each mouse was placed at three different desired points, and the time taken to find the platform was recorded at each time. If the mouse did not find the platform in 120 s, it was directed toward the platform and allowed to stay on it for 10 s. On the fifth day, the platform was removed, and a desired point was selected. Each mouse was placed at the desired point and allowed to swim for 90 s. The number of times each mouse crossed the platform was recorded, and the escape latency was recorded.

### Real-Time Polymerase Chain Reaction Analysis

Total RNA from the cerebral cortex was extracted and cDNA synthesized (Xiang et al., 2020). About 50 mg of the cerebral cortex was homogenized with TRIzol reagent (Invitrogen, California, United States) for RNA extraction. In addition, 2.5 µg of total RNA of each sample was used for cDNA synthesis using the HiFi-MMLV cDNA Kit (CoWin Biotech, Beijing, China). The transcript level was quantified by real-time PCR analysis using CFX96-Touch (Bio-Rad, California, United States) and SYBR Premix EX Taq (Takara, Otsu, Japan) under appropriate conditions. Relative gene expressions were analyzed by the 2^−*ΔΔ*Ct^ method. The 18S primer was used as internal control, and the primer sequences used are listed in Supplementary Table S1.

### Western Blot Analysis

Western blot analysis was performed on the basis of a previous report (Li et al., 2016). Briefly, about 50 mg of cerebral cortex, one hippocampus, or 50 mg of small intestine was homogenized in 1% protease and 1% phosphatase inhibitor–containing buffer, and the protein concentration was determined. In addition, about 30 µg of protein of samples was transferred to a new tube for denaturation at 100°C for 20 min. Furthermore, 30 µg of protein of each sample was loaded in each well of sodium dodecyl sulfate–polyacrylamide gel. Gel electrophoresis was run at 80 V for 15 min and 120 V for 60 min. The protein on the gel was transferred to a polyvinylidene difluoride membrane and then blocked with 5% non-fat dry milk buffer for 60 min at room temperature. The membrane was incubated with primary antibodies to TLR4 (Beijing 4A Biotech Co., Ltd., Beijing China), IKBα (Cell Signaling Technology, Boston, United States), pIKBα, occludin, ZO-1 (Santa Cruz Biotechnology, Texas, United States), and GADPH (Beijing CoWin Biotech Company, Beijing, China) at 4°C overnight. After washing the membrane three times with Tris-buffered saline, the membrane was incubated with secondary antibody (HRP-linked anti-mouse or anti-rabbit IgGs, Beijing CoWin Biotech Company, Beijing, China) for 45 min. Then, the protein bands were developed with the ECL Western blot chemiluminescence detection kit, and the blot density was measured using ImageJ software (National Institutes of Health, Bethesda, MD, United States).

### NeuN and Iba-1 Immunostaining

At the end of the animal experiment, three mice from each group were anesthetized through intraperitoneal injection of 10% chloral hydrate (0.35 ml/100 g) and perfused transcardially with cold PBS followed by cold 4% PFA dissolved in PBS. The brain was excised and fixed in 4% PFA solution overnight, then dehydrated in 15% sucrose in PBS for 24 h, and finally transferred into 30% sucrose solution for 48 h. During brain sectioning, each brain was embedded in an optimal cutting temperature compound. A 20-µm-thick brain section was cut by a cryostat (Thermo Fisher, Massachusetts, United States) and placed in a well containing antifreeze (glycerin, ethylene, and PBS in a 3:3:4 ratio). The brain sections were then stored in a refrigerator at −30°C. During immunohistochemical staining, the brain sections were fixed on a slide hydrated with PBS for 5 min. Then, they were exposed to blocking buffer for 1 h followed by 1:1000 diluted primary antibodies (NeuN or Iba-1, Abcam, Cambridge, United Kingdom) and incubated overnight at 4°C. After incubation, the sections were washed three times with PBS and incubated with 1:1000 diluted secondary antibody (Alexa Fluor 488-linked goat antirabbit IgG, Abcam, Cambridge, United Kingdom) for 2 h at 37°C. The sections were covered with DAPI and covered carefully with a coverslip. The slides of the brain section were checked with an upright two-photon confocal microscope (Olympus BX61, Shinjuku, Japan), while those with Iba-1 antibody were checked with a fluorescent microscope (Leica DMI 3000 B, Wetzlar, Germany). The neurons in the hippocampus and cerebral cortex and microglia in the cerebral cortex were counted with ImageJ software.

### Gut Microbiota Analysis

At the ninth week of experiment, fresh fecal samples of each mouse were collected and stored in liquid nitrogen until use for gut microbiota analysis. Gut microbiota analysis was performed according to a previous study (Xiang et al., 2020). Briefly, the total genomic DNA was extracted using the TIANamp Bacterial DNA Kit **(**DP302-02, Tiangen, Beijing, China), and V4 regions of the 16S rRNA gene were amplified with composite sense and antisense primers. The appropriate PCR condition was followed, and the broken sticky end of the target amplicon fragment was repaired by Klenow DNA polymerase, T4 DNA polymerase, and T4 PNK. Magnetic beads were used to purify amplicons, and replicate PCRs were pooled. Furthermore, sequence analysis was performed with UPARSE software. The sequence with ≥97% similarity was clustered for all samples, and OTUs were assigned for each representative sequence. This work was finished by LC-Bio Technology Co., Ltd., Hangzhou, China.

### Enzyme-Linked Immunosorbent Assay

At the end of experiment, the blood samples of each mouse were collected from the orbital sinus by using a capillary tube. The samples were allowed to stand at room temperature for 2 h, centrifuged at 12,000 × *g* for 15 min at 4°C to obtain the plasma, and stored in a refrigerator at −30°C until use for biochemical analysis. In addition, cerebral cortex samples were homogenized, and the protein concentrations were determined as previously explained (Xiang et al., 2020). However, to measure the fecal LPS concentration, about 30 mg of the fecal sample of each group was suspended in 1 ml sterile PBS in pyrogen-free tubes. It was homogenized mildly to prevent disruption of bacterial cells and centrifuged at 12,000 × g for 15 min at 4°C to obtain the supernatant. The supernatant was filtered through a 0.22-μm to 0.45-μm filter consecutively followed by inactivation for 15 min at 90°C. The supernatants of control groups and treatment groups were diluted with PBS at ratio of 1:2.5 and 1:5, respectively. The fecal LPS, LPS, TNF-α, IL-1β, and IL-6 concentrations in the serum and cerebral cortex were measured using commercially available mouse ELISA kits (LPS, Hefei Laier Biotechnology Co., Ltd., Hefei, China; TNF-α, IL-1β, and IL-6, Beijing 4A Biotech Co., Ltd., Beijing, China) according to the manufacturer’s instructions. Briefly, the sample and standard were added into the respective wells, followed by the addition of biotin conjugate, which was mixed properly and incubated for 2 h. After incubation, the wells were washed with wash buffer four times, and the HRP-streptavidin conjugate was added for 30 min and then washed with washing buffer four times. The substrate solution was added and incubated at 37°C for 15 min. The stop solution was added, and the optical density was checked with a microplate reader (BioTek, San Diego, California, United States) at 450 nm. The concentration of LPS, TNF-α, IL-1β, or IL-6 was calculated on the basis of the respective standard curve.

### Biostatistical Analysis

Data were evaluated with two-tailed multiple t-tests using GraphPad Prism 6.0 (GraphPad Prism, San Diego, United States). Gut microbiota was statistically analyzed with a Kruskal test or a Wilcoxon test of R software (version 3.1.1). The results were presented as mean ± SEM with *p* < 0.05 considered to be statistically significant.

## Results

### Gas Ameliorates Cognitive Dysfunction in Dgal-Induced AD Mice

To examine the cognitive function of mice, we designed various behavioral experiments including Y-maze and NOR tests to assess working memory and the MWM test to measure spatial long-term memory. The results of the Y-maze test are shown in Figures 1B,C. No statistical significant differences were observed in the numbers of arm entries in all groups (Figure 1B). However, the alternation of Dgal-only group was significantly reduced compared with that of the control (Figure 1C). Meanwhile, a significant increase in alternations was observed in Done 3 mg/kg (Figure 1C) and Gas treatment groups at the doses of 90 and 210 mg/kg, respectively (Figure 1C). The results of the NOR test, which also tests the working memory of mice, are displayed in Figure 1D. No statistical significant differences were observed in the object recognition index in all groups during the training phase. However, a marked reduction was observed in the object recognition index in the Dgal group compared with that in the normal control. At the same time, a significant increase was observed in the object recognition index in the Dgal + Done 3 mg/kg and Dgal + Gas treatment groups at doses of 90 and 210 mg/kg, respectively, compared with that in the Dgal group (Figure 1D). Furthermore, the MWM test was used to examine the spatial and long-term memory of mice, and the results are shown in Figures 1E–G. A significant increase in escape latency on training days 3 and 4 was observed in the Dgal + Gas group compared with that in the normal control (Figure 1E). Meanwhile, the escape latency of the Dgal + Gas treatment group at a dose of 90 mg/kg was significantly reduced on trainings day 3 and 4 (Figure 1E). The significant reduction of escape latency in the Done 3 mg/kg and Gas 210 mg/kg groups was observed only on training day 4 (Figure 1E). In the test phase after training for 4 days, the same changes on escape latency of each group on test day as those on training day 4 were observed (Figure 1F). By contrast, the numbers of platform crossing in the Dgal-only group was obviously decreased compared with those in the normal control (Figure 1G). However, a significant increase in platform crossing numbers was observed in the Dgal + Done and Dgal + Gas treatment groups at doses of 90 and 210 mg/kg, respectively (Figure 1G). These results suggest that we successfully constructed the aging animal model with Dgal, while Gas and Done can improve working and long-term memory of AD mice.

### Gas Changes the Gut Microbiota Composition in Dgal-Induced AD Mice

**FIGURE 2:**
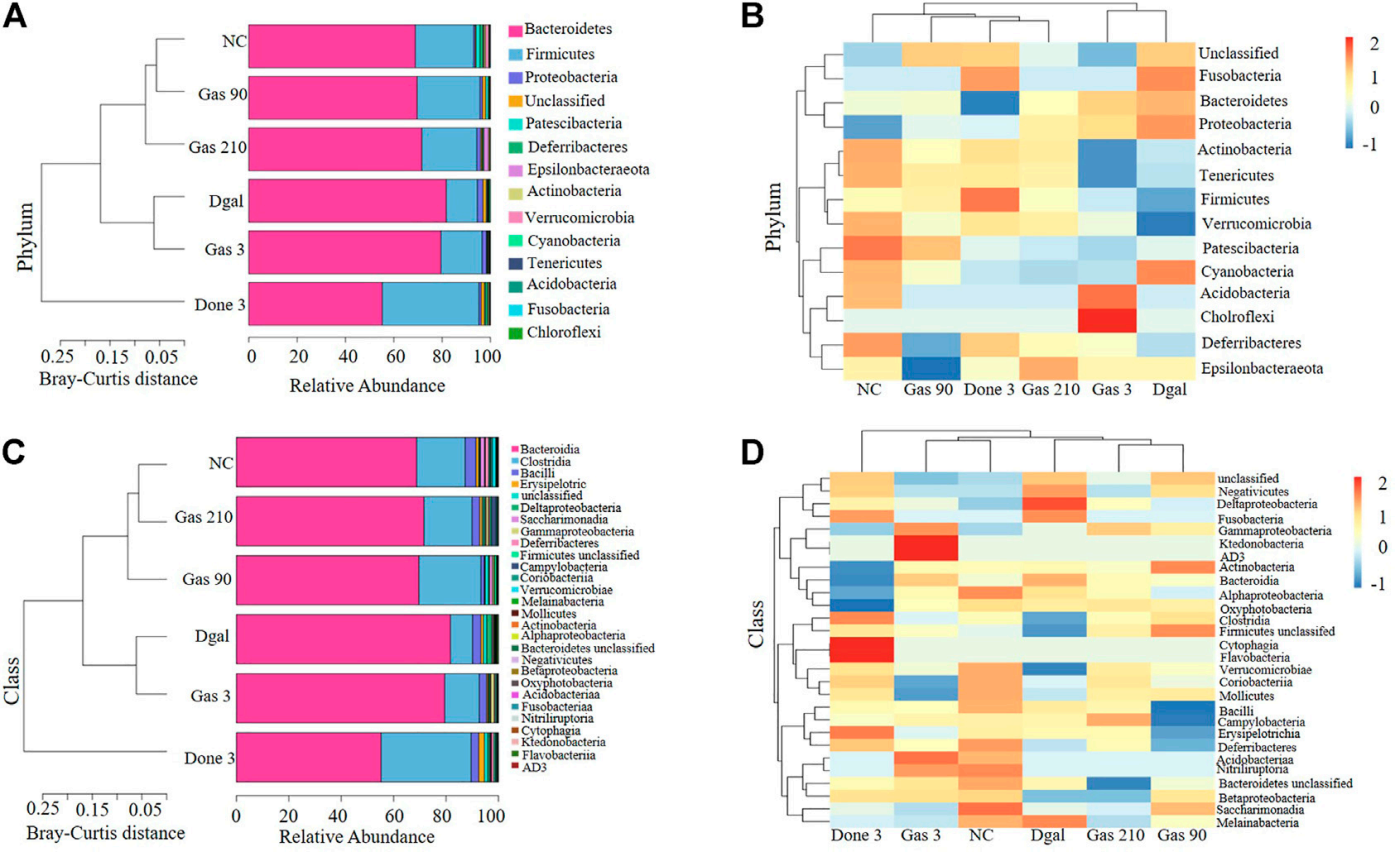
Gas changes the gut microbiota composition in AD mice. **(A)** Bray–Curtis dissimilarity cluster analysis at the phylum level. **(B)** Heat map analysis at the phylum level. **(C)** Bray–Curtis dissimilarity cluster analysis at the class level. **(D)** Heat map analysis at the class level.

To understand how Dgal and Gas affect gut microbiota, Illumina high-throughput sequencing was conducted to read the 16S rRNA sequences of the V4 region of 36 fecal microbiota samples of the normal control; Dgal, Dgal +3 mg/kg Done, and Dgal + Gas treatment groups at doses of 3, 90, and 210 mg/kg, respectively. We used Bray–Curtis dissimilarity distance to assess overall diversity in gut microbiome composition. The gut microbiome composition of the normal control and Dgal group was vastly different from each other. However, the gut microbiome composition of the Dgal +3 mg/kg Gas group was not different from that of the Dgal-only group, while the gut microbiome composition of the Dgal +90 and 210 mg/kg Gas groups was close to that of the normal control group (Figures 2A,C). Interestingly, the gut microbiota of the Dgal + Done group was significantly different from that of other groups. Thus, these results suggested that gut dysbiosis was reversed by Gas at doses of 90 and 210 mg/kg. To explore the effect of Gas on the relative abundance of microbes in the gut, we analyzed the relative abundance at the phylum and class levels. The results of phylum analysis are displayed in Figures 2A,B. A significant decrease in Tenericutes and Verrucomicrobia and a marked increase in Bacteroidetes and unclassified were observed in the Dgal group compared with that in the normal control group. Furthermore, a marked increase in the relative abundance of Firmicutes and Verrucomicrobia and significant reduction in Bacteroidetes were observed in the Dgal + Done 3 mg/kg group compared with those in the Dgal group. However, treatment with Dgal +3 mg/kg of Gas induced a significant decrease in the unclassified group compared with the Dgal group*.* After administration of Gas at doses of 90 and 210 mg/kg, a significant increase in Firmicutes and Verrucomicrobia was observed in these groups compared with that in the Dgal group. In addition, the class analysis displayed in Figures 2C,D showed a significant increase in abundance of Bacteroidia, unclassified class, and Negativicutes and a significant decrease in class Clostridia, Verrucomicrobiae, Coriobacteriia, and Mollicutes in the Dgal group compared with that in the normal control. However, treatment with 3 mg/kg of Done significantly increased the abundance of Clostridia, Verrucomicrobiae, and Firmicutes unclassified and decreased that of Bacteroidia and Negativicuts compared with that in the Dgal group. A significant decrease in the abundance of Negativicutes in all Gas groups, unclassified class in the 3 and 210 mg/kg Gas-treated group, and Erysipelotrichia in the 90 mg/kg Gas group was observed compared with that in the Dgal group. Meanwhile, a significant increase in Clostridia in the 90 mg/kg Gas group and Gammaproteobacteria, Coriobacteriia, and Verrucomicrobiae in the 210 mg/kg Gas group was observed compared with that in the Dgal group. These results indicated that Gas significantly increased the relative abundance of Firmicutes and Verrucomicrobiae at the phylum and class levels*.*

### Gas Promotes Neuroprotection and Mitigates Microglial Activation in Dgal-Induced AD Mice

**FIGURE 3:**
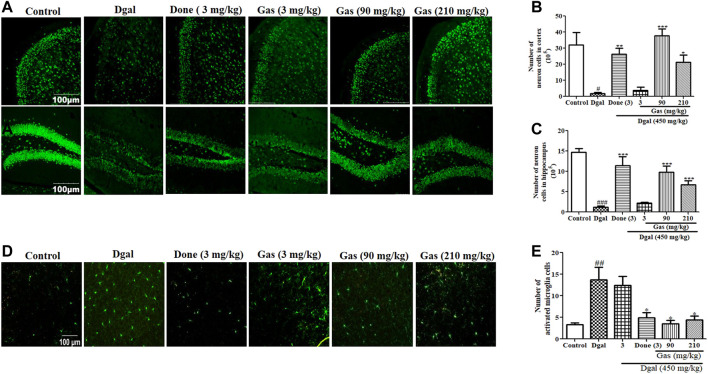
Gas promotes neuroprotection and mitigates microglial activation in Dgal-induced AD mice. **(A)** Fluorescent image of neurons in the hippocampus and cerebral cortex of all groups. **(B)** and **(C)** represent digital number of neurons in the cerebral cortex and hippocampus, respectively. **(D)** Fluorescent image of microglia in the cerebral cortex. **(E)** Digital number of microglia in the cerebral cortex. The brains of three mice in each group were cut, and six sections of the cerebral cortex and hippocampus of each mouse were used to calculate neurons or microglia. The data were presented as means ± SEM. ^##^ and ^###^ indicate significant differences at *p* < 0.01 and *p* < 0.001 compared with those of the normal control, respectively; ^*, **, and ***^ indicate significant difference at *p* < 0.05, *p* < 0.01, and *p* < 0.001 compared with those of the Dgal group, respectively.

To determine whether Gas exerts neuroprotective effects and affects the activity of microglia in the brain, we used immunohistochemical staining to detect the changes of neurons in the cerebral cortex and hippocampus of the brain. The photographs and digitally calculated number of neurons in the cerebral cortex and hippocampus are displayed in Figures 3A–C. The number of neurons in the cerebral cortex and hippocampus was significantly decreased in the Dgal group compared with that in the normal control group. No clear change in the number of neurons was observed in the cerebral cortex and hippocampus of the D-gal + 3 mg/kg Gas treatment group compared with that in the Dgal group. However, the Dgal + Done group and Dgal + Gas 90 and Dgal +210 mg/kg groups had significantly more neurons in the cerebral cortex and hippocampus than the Dgal group (Figure 3B). Gas at a dose of 90 mg/kg showed the best neuroprotective effect. Subsequently, we detected the changes of microglia in the cerebral cortex of the brain, and the results are shown in Figures 3D,E. The numbers of microglia in the cerebral cortex were significantly higher in the Dgal group (*p* < 0.01). Nevertheless, the numbers of microglia in the cerebral cortex of the Dgal + Done group and all Dgal + Gas groups were obviously lower than those of the Dgal alone group. Furthermore, we assessed BDNF gene expression in the cerebral cortex of mice. A marked decrease of BDNF expression in the Dgal group and evident increase of BDNF expression in the Dgal + Done and Dgal + Gas treatment groups at doses of 90 and 210 mg/kg were observed compared with those in the normal control and Dgal group (Supplementary Figure S1A). The results indicated that Gas improved the memory of AD mice *via* modifying neuroprotection and decreasing microglia, and the dose of 90 mg/kg was best among the tested concentrations.

### Gas Mitigates Serum Inflammation and Neuroinflammation in Dgal-Induced AD Mice

**FIGURE 4:**
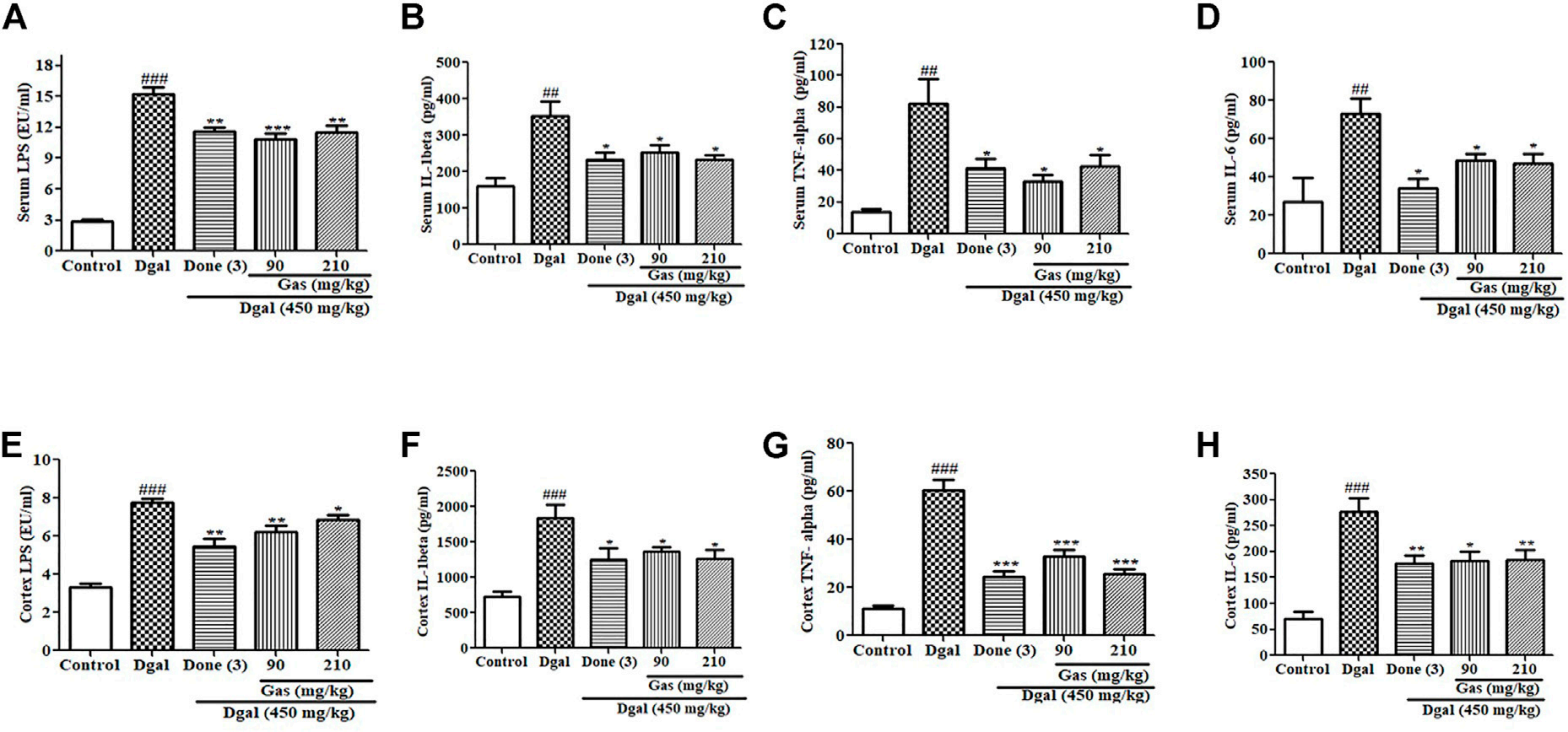
Effects of Gas on proinflammatory cytokines in the serum and cerebral cortex. **(A–D)** Changes in the serum LPS, IL-1β, TNF-α, and IL-6 of AD mice after treatment with Gas, respectively. **(E–H)** Concentration of cerebral cortex LPS, IL-1β, TNF-α, and IL6. Sample numbers are five, and the data are presented as means ± SEM. ^#, ##,^ and ^###^ indicate significant differences at *p* < 0.05, *p* < 0.01, and *p* < 0.001 compared with those in the normal control, respectively; ^*, **, and ***^ indicate significant difference at *p* < 0.05, *p* < 0.01, and *p* < 0.001 compared with those of the Dgal group, respectively.

Gut dysbiosis has been implicated in promoting increased LPS levels in the blood, which induces serum inflammation and promotes neuroinflammation (He et al., 2021). In this study, we investigated the signaling pathway of inflammation. The fecal LPS and plasma LPS in the Dgal group were significantly higher than those in the normal control (Supplementary Figure S5D, Figure 4A). After drug treatment, the fecal LPS and plasma LPS levels in the Dgal + Done and Dgal + Gas 90 and 210 mg/kg groups were lower than those in the Dgal group (Supplementary Figure S5D, Figure 4A). Furthermore, we measured the proinflammatory cytokines in the plasma, and the results are displayed in Figures 4B–D. Similar increase and reduction of serum IL-1β, TNF-α, and IL-6 were observed in the Dgal group and all drug treatment groups (Figures 4B–D). We examined these parameters in the brain, and the results are shown in Figures 4E–H. Increased cerebral cortex LPS in the Dgal group and decreased cerebral cortex LPS in the Dgal + Done and Dgal + Gas groups at doses of 90 and 210 mg/kg were observed compared with those in the normal control and Dgal group (Figure 4E). The proinflammatory cytokines, such as IL-1β, TNF-α, and IL-6 in the brain were significantly increased in the Dgal group and decreased in the drug treatment groups (Figures 4F,H). These results revealed that Gas can mitigate neuroinflammation of AD mice induced by Dgal *via* attenuating endotoxin and proinflammatory cytokines in the plasma and brain.

### Gas Ameliorates Neuroinflammation Through the TLR4/NF-κB Pathway in Dgal-Induced AD Mice

**FIGURE 5:**
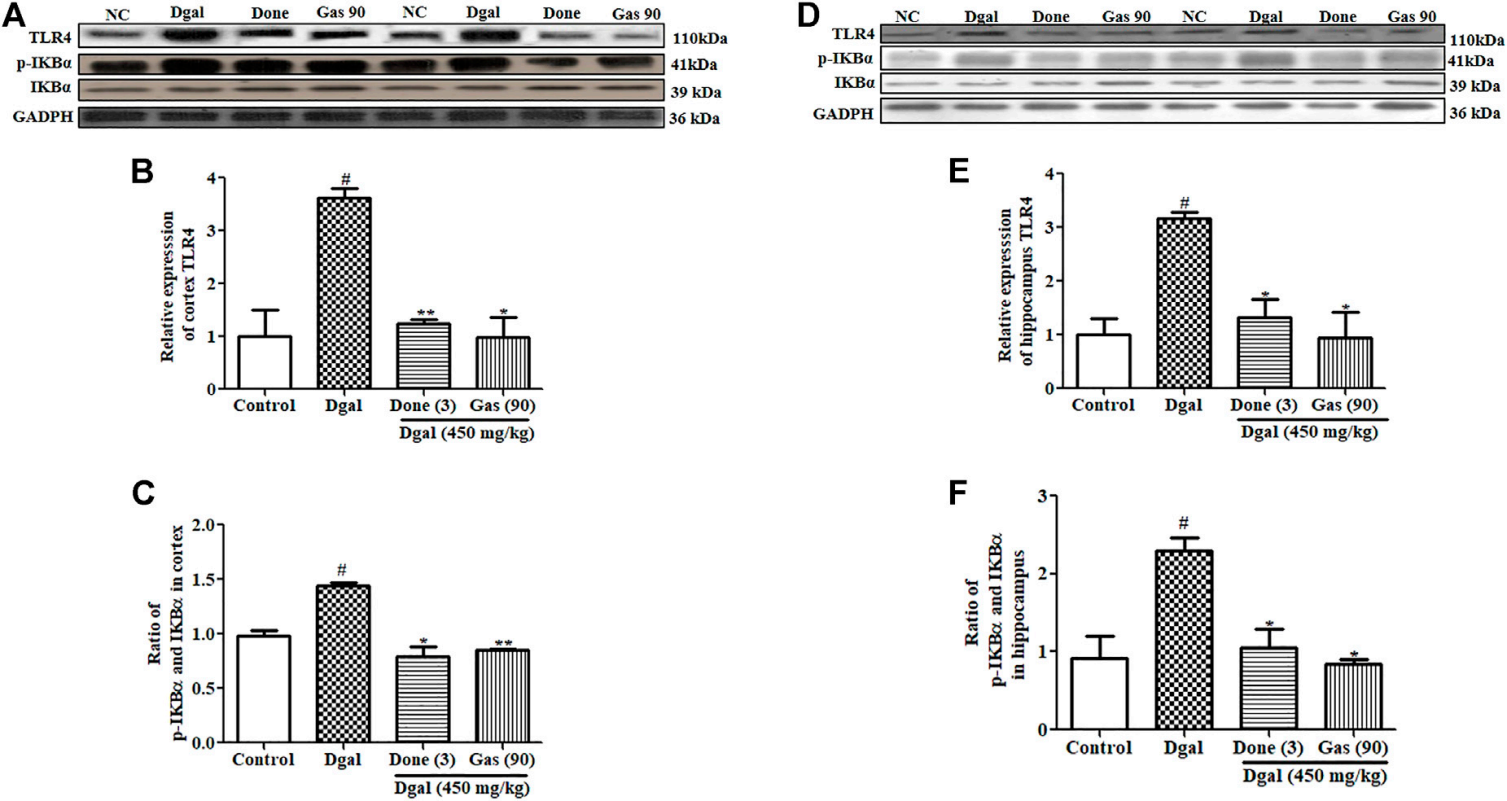
Effect of Gas on the TLR4/NF-κB signaling pathway in the cerebral cortex and hippocampus. **(A)** Western blot analysis of the cerebral cortex TLR4, pIKBα, and IKBα. **(B)** and **(C)** represent digitalized results of Western blot analysis. **(D)** Western blot analysis of TLR4, pIKBα, and IKBα in the hippocampus. **(E)** and **(F)** represent digitalized Western blot analysis of TLR4, pIKBα, and IKBα in the hippocampus, respectively. Sample number of each group is three, and the data are presented as means ± SEM. ^#^ indicates significant differences at *p* < 0.05 compared with that in the normal control; ^* and **^ indicate significant difference at *p* < 0.05 and *p* < 0.01, respectively, compared with those in the Dgal group.

An increase in LPS has been shown to activate the TLR4 signaling pathway, which increases the expression of proinflammatory markers (Jamar et al., 2021). We considered that LPS might activate the TLR4/NF-κB signaling pathway in the brain of mice. Thus, we investigated the changes of this signaling pathway in the cerebral cortex and hippocampus. A marked increase in TLR4 and phosphorylated IκBα protein levels was observed in the cerebral cortex and hippocampus of the Dgal group compared with that in the normal control (Figures 5A–F; Supplementary Figure S2A,B). At the same time, these patterns in the cerebral cortex and hippocampus were significantly decreased in the Dgal + Done and Dgal + Gas treatment groups at a dose of 90 mg/kg (Figure 5, Supplementary Figure S2A,B). In addition, NF-κB and IKKβ gene expression was significantly increased in the Dgal group compared with that in the normal control (Supplementary Figure S1B,C) but was significantly reduced in the Dgal + Done and Dgal + Gas treatment groups at a dose of 90 and 210 mg/kg, respectively (Supplementary Figure S1B,C). These results revealed that Gas reduced neuroinflammation in the brain of AD mice by regulating the TLR4/NF-κB signaling pathway.

### Gas Upholds the Integrity of the Intestinal Barrier and Blood–Brain Barrier (BBB)

**FIGURE 6:**
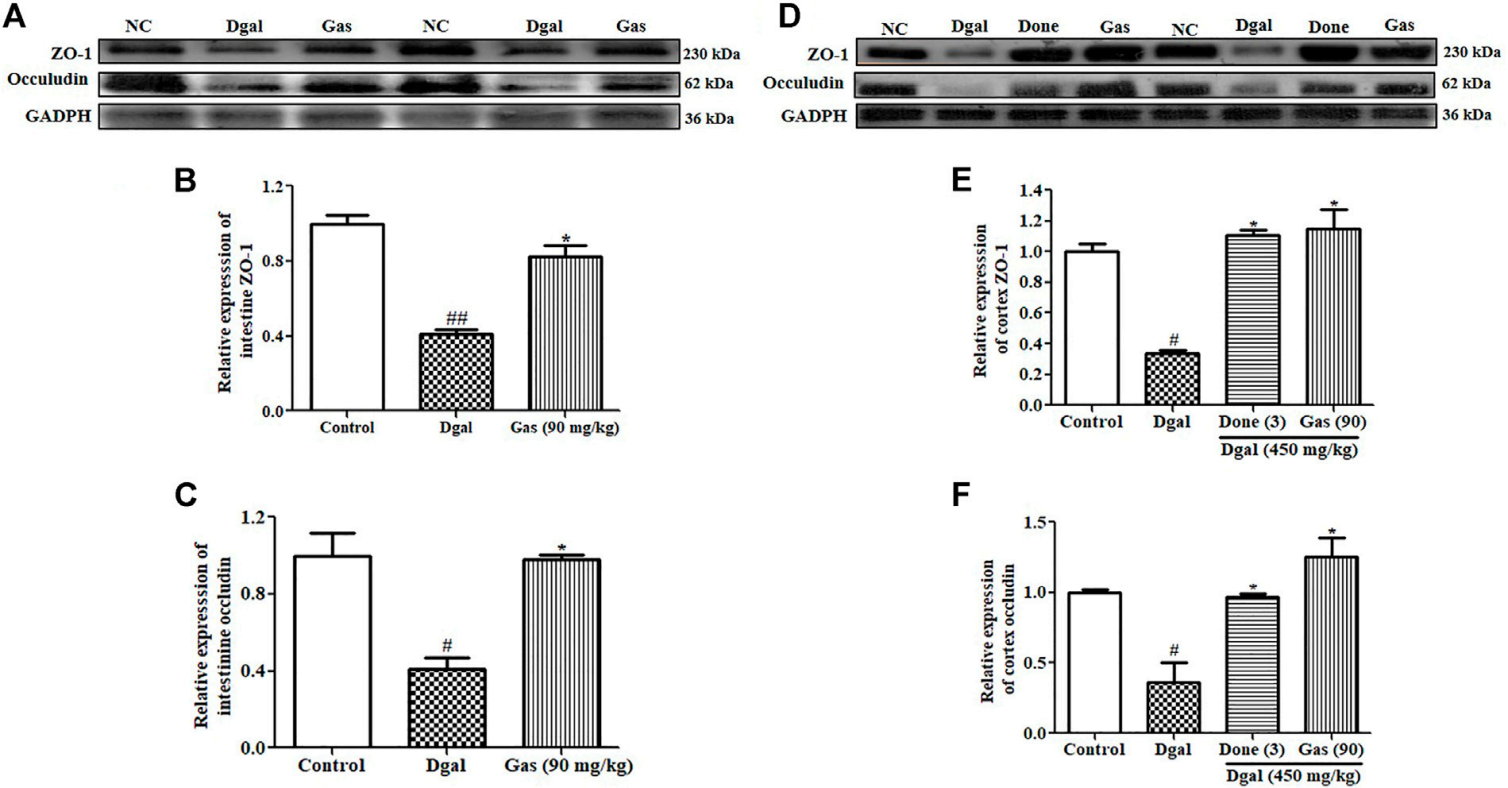
Gas upholds the integrity of the intestinal barrier and BBB. **(A)** Western blot analysis of the small intestine ZO-1 and occludin. **(B)** and **(C)** represent digitalized Western blot results of ZO-1 and occludin in the small intestine. **(D)** Western blot analysis of the cerebral cortex ZO-1 and occludin. **(E)** and **(F)** represent digitalized Western blot results of the cerebral cortex ZO-1 and occludin. Sample number of each group is three, and the data are presented as means ± SEM. ^# and ##^ indicate significant differences at *p* < 0.05 and *p* < 0.01 compared with those of the normal control, respectively; ^*^ indicates significant difference at *p* < 0.05 compared with that in the Dgal group.

The intestinal barrier and BBB are important in preventing microorganism invasion into blood circulation and the brain. Intestinal tight junction leakage increases LPS and bacteria in the blood (Jamar et al., 2021). Brain tight junction leakage has been implicated in the progression of neurodegenerative diseases (Varatharaj and Galea, 2017). Thus, we explored two important tight junction proteins, namely, zonulin and occludin, and the results are shown in Figure 6. The zonulin and occludin proteins of the intestine were significantly decreased in the Dgal-only group (Figures 6A–C, Supplementary Figure S2C) compared with those in the normal control. After administering Gas at 90 mg/kg, zonulin and occludin protein expression levels in the intestine were significantly increased compared with those in the Dgal group (Figures 6A–C; Supplementary Figure S2C). As we expected, the changes of zonulin and occludin in the brain were similar to what is observed in the intestine (Figures 6D–F, Supplementary Figure S2D). These findings suggest that Dgal damaged the intestinal barrier and BBB of Dgal-induced AD mice, and Gas ameliorated these damages and promoted neuroprotection.

### Gas Promotes Cognition and Neuroprotection Partly by Regulating Gut Microbiota Composition

**FIGURE 7:**
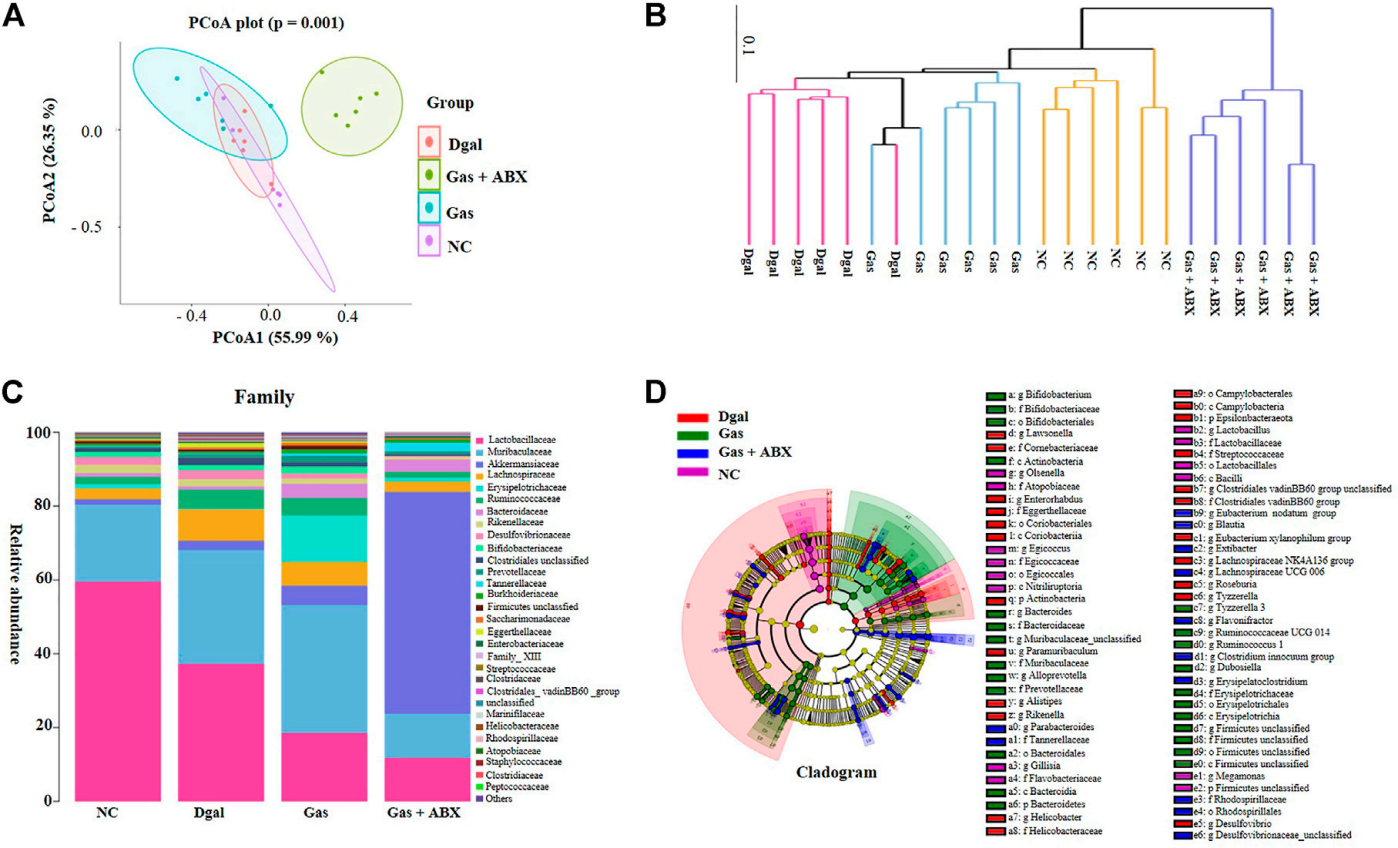
Effects of Gas and antibiotics cocktail on the gut microbiota composition of AD mice. **(A)** PCoA of gut microbiota in all groups. **(B)** UPGMA tree of gut microbiota in all experimental groups. **(C)** Stacked bar chart of the relative abundance of gut microbiota at the family level. **(D)** Cladogram of gut microbiota in all groups.

To determine whether gut microbiota is involved in the neuroprotection of Gas, we administered an antibiotic cocktail to mice for 2 weeks to deplete the gut microbiota of mice before administering Dgal and Gas. The gut microbiota of AD mice at the end of the animal experiment was analyzed again with 16S rRNA sequence analysis. The results of principal coordinate analysis (PCoA) based on the OTU abundance are shown in Figure 7A. The Dgal + Gas + ABX group and other groups could be distinguished clearly on the basis of the gut microbiota composition. Meanwhile, 90 mg/kg of Gas significantly affected the gut microbiota composition of AD mice induced by Dgal (Figure 7A). The results of multivariate ANOVA of the PCoA matrix scores indicated a statistically significant difference between the microbiota of the Dgal + Gas + ABX group and other groups. Significant differences were also observed in the normal control, Dgal, and 90 mg/kg Gas groups (Figure 7B). To understand how Gas and ABX affected the gut microbiota of AD mice, we further focused on family-level analysis, and the results are shown in Figure 7C. Dgal significantly increased the abundance of the Clostridiales_vadinBB60_ group, Staphylococcaceae, and Marinifilaceae and reduced that of Erysipelotrichaceae, Atopobiaceae of gut microbiota in mice. A significant increase in the abundance of Erysipelotrichaceae, Bacteroidaceae, Rhodospirillaceae, Tannerellaceae, and Atopobiaceae and significant decrease in the abundance of the Clostridiales_vadinBB60_ group and Helicobacteraceae were observed in the Gas treatment groups compared with those in the Dgal group. Meanwhile, ABX cocktail significantly reduced the abundance of Bifidobacteriaceae, Eggerthellaceae, Atopobiaceae, Muribaculaceae, Staphylococcaceae, Peptococcaceae, Marinifilaceae, Firmicutes_ unclassified, Streptococcaceae, and Erysipelotrichaceae and significantly increased the abundance of Akkermansiaceae, Tannerellaceae, Enterobacteriaceae, and Rhodospirillaceae compared with those in the Gas treatment group. These results demonstrated that Gas may increase the relative abundance of cognition beneficial microbes, such as Bacteroidaceae, Muribaculaceae, and Erysipelotrichaceae. Meanwhile, ABX significantly reduced these beneficial microbes for memory and significantly increased Akkermansiaceae to diminish the neuroprotective function of Gas.

To compare the gut microbial community composition, a logarithmic LDA score cutoff of 3 and profiling of LDA effect size analysis were used to identify important taxonomic differences between groups. In the normal control group, the significantly different microbiota was Olsenella, Atopobiaceae, Egicoccus, Egicoccales, Nitriliruptoria, *Lactobacillus*, Lactobacillaceae, Lactobacillales, Bacilli, Firmicutes, Flavobacteriaceae, Megamonas, Gillisia, and Xanthomonadaceae unclassified. Meanwhile, the abundance of Actinobacteria, Desulfovibrio, Coriobacteriia, Epsilonbacteraeota, Roseburia, Rikenella, Helicobacter, Anaerotignum, and Clostridiales vadinBB60 group showed a significant difference in the Dgal group. After administering Gas, a significant difference was observed in the abundance of Bacteroidetes, Muribaculaceae, Erysipelotrichales, *Lactobacillus*, Ruminococcaceae UCG 014, Actinobacteria, Bifidobacterium, Firmicutes, and Ruminococcus in the Gas treatment groups. Interestingly, significant differences were observed in Akkermansiaceae, Verrucomicrobia, Parabacteroides, *Bacteroides caecimuris*, Blautia, Enterobacteriaceae, *Clostridium innocuum*, Desulfovibrionaceae, Eubacterium nodatum group, Insolitispirillum, Lachnospiraceae UCG 006, Erysipelatoclostridium, Parasutterella, Extibacter, Flavonifractor, and Enterobacteriaceae in the ABX-treated group (Figure 7D, Supplementary Figure S3). These results demonstrated that Gas induces the proliferation of cognition beneficial microbes, such as Muribaculaceae, Erysipelotrichales, and Bifidobacterium, while ABX significantly reduced beneficial microbes for memory and significantly increased Akkermansiaceae to offset the effects of Gas.

### ABX Partially Diminishes the Neuroprotective Effect of Gas *via* the Interference of Gut Microbiota

**FIGURE 8:**
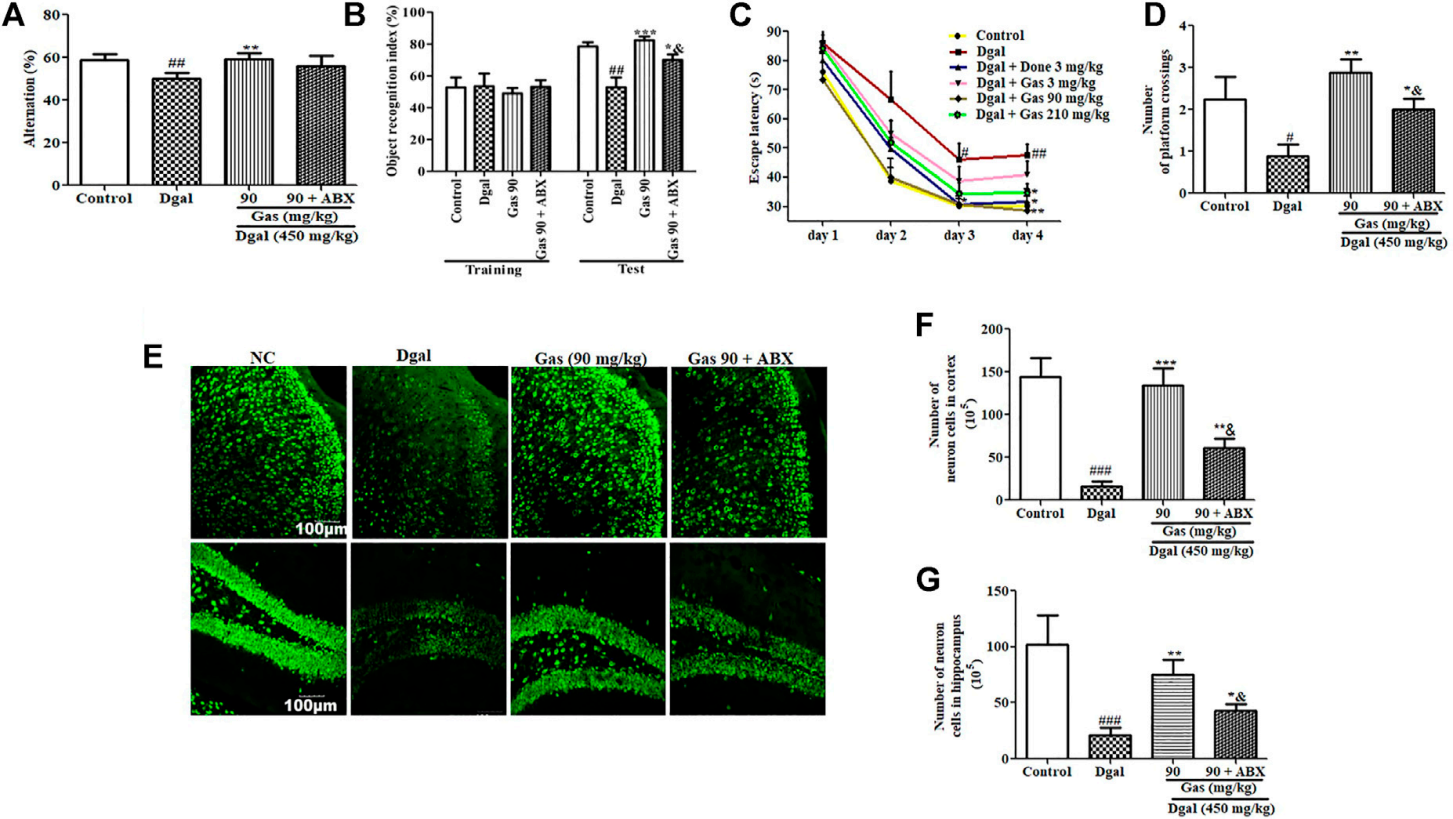
Antibiotic cocktail partially diminishes the neuroprotective effect of Gas *via* the interference of gut microbiota. **(A)** Changes of alternation in the Y-maze test. **(B)** Object recognition index in the NOR test. **(C)** Escape latency in the training phase in the MWM test. **(D)** Number of platform crossings in the test phase in the MWM experiment. **(E)** Fluorescent image of the cerebral cortex and hippocampus neurons of AD mice after treatment with Gas. **(F,G)** Digitalized results of hippocampus and cerebral cortex neurons. The brains of three mice in each group were cut, and six sections of the cerebral cortex and hippocampus of each mouse were used to calculate the neuron. The data are presented as means ± SEM. The data were presented as means ± SEM. ^#, ##,^ and ^###^ indicate significant differences at *p* < 0.05, *p* < 0.01, and *p* < 0.001 compared with those of the normal control, respectively; ^*, **, and ***^ indicate significant difference at *p* < 0.05, *p* < 0.01, and *p* < 0.001 compared with those in the Dgal group, respectively. ^&^ represents significant difference at *p* < 0.05 compared with that of the Gas group.

Furthermore, we performed animal behavioral experiments to assess changes in cognition of AD mice after administering the antibiotic cocktail. Significant reduction of alternation in the Y-maze test and object recognition index in the NOR test was observed in the Dgal group. Meanwhile, these parameters were recovered in the 90-mg/kg Gas treatment group (Figures 8A,B, Supplementary Figure S4A). Furthermore, the increase of training day 4 escape latency and reduction of platform crossing numbers in the MWM test were observed in the Dgal group compared with those in the normal control (Figures 8C,D, Supplementary Figure S4B). Meanwhile, these parameters were regained in the 90-mg/kg Gas treatment group (Figures 8C,D, Supplementary Figure S4B). As we expected, the increase of these parameters in the 90-mg/kg Gas treatment group were offset or partially offset by antibiotic cocktail (Figures 8A–D, Supplementary Figure S4A–B). Moreover, we performed tissue biopsies and immunostaining of the brain to detect changes in the cerebral cortex and hippocampus of AD mice after administering ABX. The significant reduction of neurons in the cerebral cortex and hippocampus was observed in the Dgal group (Figures 8E–G) and 90-mg/kg Gas treatment suppressed the reduction (Figures 8E–G). However, treatment with ABX significantly reduced neurons in the cerebral cortex and hippocampus compared with the Gas treatment groups (Figures 8F,G). Interestingly, ABX did not completely eliminate the effects of Gas. These results suggested that gut microbiota partially contribute in neuroprotection.

## Discussion

*Gastrodia elata* is a Chinese herbal medicine used for treating neurodegenerative diseases, such as AD and Parkinson’s disease (Jang et al., 2015). Gas is the main constituent of *G. elata* (Liu et al., 2018) and has been reported to have antioxidative, anticancer, anticonvulsive, and neuroprotective properties (Liu et al., 2018). In spite of Gas being reported to improve the memory of AD mice such as APP/PS1 and Tg 2576 (Virgili et al., 2018; Liu et al., 2018; Hu et al., 2014), the detailed mechanism of action of Gas remains unclear. Growing evidence shows that the gut–brain axis is a pharmacological target for cognitive impairments. Therefore, in this study, we used Dgal to induce AD in mice and investigated the mechanism of action of Gas *via* the gut–brain axis. The results of animal behavior experiments in Figures 1B–G suggested that we successfully constructed AD model mice with Dgal, and Done and Gas at doses of 90 and 210 mg/kg, respectively, could improve the memory of AD mice. Our results are consistent with those of other studies (Obrenovich et al., 2020; Zeng et al., 2021)

Recently, other studies have reported that the gut–brain axis plays a vital role in brain functions, making the gut microbiota an emerging pharmacological target for brain aging (Kowalski and Mulak, 2019; Sharma et al., 2021). To elucidate the effect of Gas on gut microbiota, we used the 16S rRNA gene sequencing technology. The increase of Bacteroidetes and reduction of Tenericutes and Verrucomicrobia in the Dgal group and significant increase of the relative abundance of Firmicutes and Verrucomicrobiae after administering Gas (Figures 2A–D) indicated that Dgal induced gut microbiota dysbiosis, and Gas administration reversed it.

In the central nervous system, the hippocampus and cerebral cortex have important roles for memory. The hippocampus is involved in memory formation and storage of short-term memory traces, meanwhile the cortex took part in long-term storage of memory (Gräff et al., 2012). Moreover, they produced the interaction *via* modification of histone posttranslation. The hippocampus transferred memory traces to the cortex for memory consolidation and long-term storage and had feedback interactions for memory retrieval (Gräff et al., 2012; Preston and Eichenbaum, 2013)**.** Therefore, we detected changes in neurons of the cerebral cortex and hippocampus with immunostaining. As we expected, the significant reduction of the cortex and hippocampus neurons in the Dgal group was observed and the neuron-decrease was fixed by Done and Gas administration (Figures 3A–C). The observations shown in Figures 1–3 suggested that the gut microbiome is involved in the neuroprotective effects of Gas and Done. In the present study, we found that Gas has neuron protection effects on both the cortex and hippocampus. The results of the animal behavior experiment (Figure 1and Figure 8) indicated that Gas can improve the short-term memory and long-term storage of memory. We will focus of Gas effect on modification of histone posttranslation in future studies.

Neuroinflammation was a design feature of central nervous system disease and was recognized as a potential mediator of cognitive impairments. Systemic inflammation levels are increased with advanced age and neurodegeneration. The impact of age on neuroinflammatory responses including glial activation, increase of proinflammatory cytokines, and aberrant neuronal signaling could lead to the worse of the central nervous system microenvironment in disease and acceleration of cognitive impairment (Kumar, 2018). Furthermore, pathological increase in proinflammatory cytokines in the brain promoted neuron inflammation that caused neuronal death and cognitive dysfunction (He et al., 2021). Dgal induced gut dysbiosis, which increases LPS levels in blood (He et al., 2021). Gut dysbiosis affects intestinal integrity by promoting a leaky gut, which causes LPS to enter the blood circulation (He et al., 2021). This process induces systemic inflammation, which reaches the brain and promotes neuroinflammation by activating the TLR4 signaling pathway (Liu et al., 2019). In the present study, we focused on the integrity of the intestinal barrier and BBB; LPS of the plasma, cortex, and hippocampus; and TLR4/NF-κB signaling pathway to investigate the mechanism of action of Gas. The changes in these parameters in Figures 4–6 and Supplementary Figure S5D revealed that Gas improved the memory of Dgal-induced AD mice by preventing LPS produced in the intestine to enter the blood and brain, reducing proinflammatory cytokines and mitigating the TLR4/NF-κB signaling pathway. Furthermore, we investigated the effect of Gas on microglia in the cortex. The results in Figures 3D,E suggested that microglia are involved in the neuroprotective effect of Gas.

The reduction of tight junction proteins, such as zonulin and occludin in Gal-induced AD mice were observed in this study. However, the downregulation of tight junction proteins does not necessary imply loss of function of the barriers. It is merely an indirect pointer and does not imply the loss of function of the intestine barrier and blood–brain barrier. In the future study, we will select phenolphthalein excretion test and circulating D-lactic acid assay to measure the permeability of the intestinal barrier and use the time-domain optical near-infrared imaging to evaluate the permeability of the blood–brain barrier of AD mice to confirm whether the intestinal barrier and BBB take important roles during neuroprotection of Gas.

To obtain direct evidence to support our hypothesis that the gut–brain axis plays an important role in the neuroprotective effect of Gas, we used antibiotic cocktail to deplete gut microbiota of mice and examine the effect of Gas on the gut microbiota and memory of AD mice again. The significant changes on gut microbial composition of AD mice (Figure 7), reduction of alteration in Y-maze and NOR index in NOR tests (Figures 8A,B), reduction of neurons in the cerebral cortex and hippocampus (Figures 8D–F), and increase of escape latencies and reduction of crossing numbers of the platform in the MWM experiment (Figures 8C,D) after administering antibiotics indicated that the gut–brain axis plays a vital role in the neuroprotective effect of Gas. However, the effect of Gas on the memory of AD mice was not completely diminished by antibiotics. This evidence illustrated that Gas not only targets the gut microbiota but also directly enters the blood circulation, traverses the BBB, and arrives in the cerebral cortex and hippocampus to exert neuroprotection.

In the previous studies, Rikenellaceae, Desulfovibrio, Cyanobacteria, Proteobacteria, Deferribacter, Helicobacter, and Escherichia have been indicated to be the harmful gut microbiota for cognitive function. Rikenellaceae was overrepresented in old mice, and the genus Alistipes of this family could modulate neuronal signals in anxiety (Murros et al., 2021; Wu et al., 2021). Desulfovibrio produce hydrogen sulfide, induce gut permeability, and promote toxin leakage that debilitates brain function (Murros et al., 2021). Cyanobacteria may produce the neurotoxin β-N-methylamino-l-alanine (BMAA) to promote nervous system dysfunction and increase the formation of Aβ plaques (Silva et al., 2020). Proteobacteria can induce neuroinflammation and microglia activation and promote cognitive decline (Carabotti et al., 2015; Silva et al., 2020). Deferribacter as a pathobiont increased inflammation (Carabotti et al., 2015). Helicobacter can induce several cytokines such as IL-6 and TNF-α that can contribute to BBB disruption and promote neurodegeneration (Akhmedov et al., 2009). Escherichia produces extracellular amyloid that can bind to Toll-like receptor 2 and trigger downstream inflammatory responses, which affect cognitive functions (Tükel et al., 2010). On the contrary, Ruminococcus, *Akkermansia muciniphila*, Negativibacillus, Clostridium, Enbacterium, and Odoribacter are beneficial gut microbiota for cognitive function. Ruminococcus produces butyrate, inhibits histone deacetylase, mitigates the secretion of proinflammatory cytokines, and promotes cognitive function (Jiang et al., 2021). Furthermore, the butyrate level in the plasma is negatively correlated with Aβ deposition in cognitively impaired individuals and butyrate decreases BBB permeability and IL-1β expression in LPS-induced AD mice (Marizzoni et al., 2020). *Akkermansia muciniphila* mitigates intestinal barrier dysfunction, reduces the deposition of Aβ plaques in the brain, promotes cognitive function, and delays pathological changes in the brain (Ou et al., 2020; Higarza et al., 2021). Negativibacillus has a positive correlation with cognition (Sadovnikova et al., 2021). *Clostridium* can synthesize 3-indolepropionic acid, a strong antioxidant that can scavenge free radicals produced by D-gal, thus protecting the brain from oxidative damage. It also reduces neuronal apoptosis, mitigates histopathological changes and BBB permeability, and promotes cognitive function (Tükel et al., 2010; Liu et al., 2015; Zhang et al., 2018; Obrenovich et al., 2020). Eubacteria and Odoribacter are positively correlated with cognitive function (Zhou et al., 2021). These data and the significant changes on these gut microbiotas after administering Gas in our study (Figures 2A,B, Supplementary Figure S5A,B) strongly supported our conclusion that Gas promotes beneficial gut microbes and mitigates non-beneficial ones to improve the memory of AD mice *via* the modification of the gut–brain axis.

Interestingly, the abundance of *Akkermansia muciniphila*, which produce beneficial metabolites for the brain, was significantly increased after antibiotic treatment in our study. However, the memory of AD mice was not increased by the excessive increase of *Akkermansia muciniphila.* This finding indicated that the ratio of gut microbiota is very important for optimal function. Our results were consistent with those of other studies, which reported that the overcolonization of *Akkermansia muciniphila* promotes inflammation and *Akkermansia muciniphila* is overrepresented in Parkinson’s disease patients (Seregin et al., 2017; Lubomski et al., 2021).

In the present study, we used intraperitoneal injection of Dgal to induce AD in mice. Although significant changes in gut microbiota were observed, the results of PCoA showed the overlap of gut microbiota in the normal control and Dgal groups (Supplementary Figure 5C). It is possible that the effect of intraperitoneal injection of Dgal on gut microbiota was weaker than that of oral administration. In a future study, we will compare the effect of these two methods on gut microbiota and select the best way to construct an AD model. Furthermore, we will use transgenic AD mice or natural aging mice to confirm whether Gas has the same efficacy and mechanism of action in different AD models.

On the other hand, we performed bacterial phenotypic prediction of gut microbiota in our study. The reduction of aerobic, gram-positive, and containing mobile elements and the increase of anerobic, gram-negative, and potentially pathogenic were observed in the Dgal group compared with those in the normal control (Supplementary Figure S6A–F). These parameters were reversed to the normal levels in the Done and 90 mg/kg Gas groups (Supplementary Figure S6A–F). In addition, Gas activates biosynthesis of tetrahydofolate, biotin, stearate, oleate, palmitate, palmitoleate, and lipid IVA (Supplementary Figure S7). These results confirmed that the microbiome–gut–brain axis is involved in the neuroprotective effect of Gas.

## Conclusion

**FIGURE 9:**
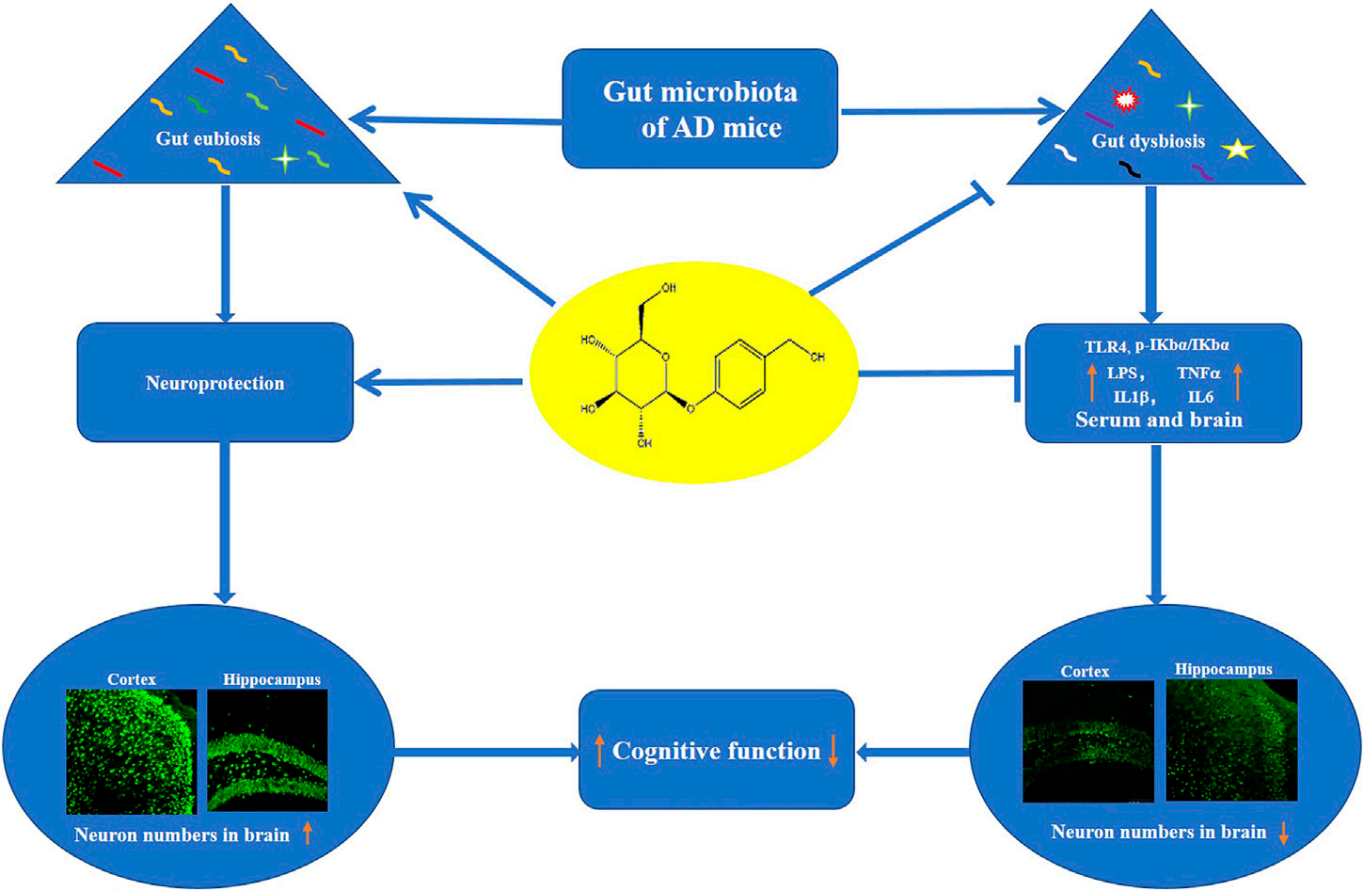
Proposed mechanism of action of Gas. Gas improved the memory of Dgal-induced AD mice *via* regulating the composition of gut microbiota and inhibition of neuroinflammation.

In conclusion, Dgal induced the dysbiosis of gut microbiota to produce LPS and impaired the integrity of the intestinal barrier and BBB. Furthermore, LPS enters the blood to induce neuroinflammation and cognitive decline. Gas improves the memory of AD mice *via* increasing abundance of *Lactobacillus* and Firmicutes, which produce GABA, acetylcholine, and histamine to promote cognitive function and reducing abundance of pathogenic bacteria. Moreover, the Gas maintained intestinal barrier and BBB function, which was damaged by Dgal treatment to protect the brain from LPS and proinflammatory cytokines (Figure 9). Our study provides a scientific basis for the development of drugs targeting gut microbes.

## Data Availability

The datasets presented in this study can be found in online repositories. The names of the repository/repositories and accession number(s) can be found below: https://www.ncbi.nlm.nih.gov/bioproject; PRJNA781105.

## Abbreviations

AD, Alzheimer’s disease; ABX, antibiotics; ANOVA, analysis of variance; BBB, blood–brain barrier; BDNF, brain-derived neurotrophic factor; Dgal, D-galactose; Done, donepezil; DAPI, 4′, 6-diamidino-2-phenylindole; GADPH, glyceraldehyde-3-phosphate dehydrogenase; ELISA, enzyme-linked immunosorbent assay; Gas, gastrodin; IL-1β, interleukin-1 beta; IL-6, interleukin 6; Iba-1 ionized calcium-binding adapter molecule; LEFSE, linear discriminant analysis effect size; LDA, linear discriminant analysis; LPS, lipopolysaccharide; MWM, Morris water maze; NEUN, neuronal nuclear protein; NOR, novel object recognition; PCoA, principal coordinate analysis; PFA, paraformaldehyde; RT-PCR, real-time polymerase chain reaction; TLR4, Toll-like receptor 4; TNF-α, tumor necrosis factor alpha; and ZO-1, zonulin.

## References

[B1] Akhmedov V. A. Kritevich M. A. Ostapenko V. A. Sokolova T. F. (2009). Dynamics of Pro-and Anti-inflammatory Cytokines at Patients with Combination of Chronic Opisthorchiasis with Helicobacter Pylori-Associated Gastritis. Eksp Klin Gastroenterol. (1), 20–25. Available at: https://pubmed.ncbi.nlm.nih.gov/19551959 . 19551959

[B2] Akhtar A. Sah S. P. (2020). Insulin Signaling Pathway and Related Molecules: Role in Neurodegeneration and Alzheimer's Disease. Neurochem. Int. 135, 104707. 10.1016/j.neuint.2020.104707 32092326

[B3] Ali A. Shah S. A. Zaman N. Uddin M. N. Khan W. Ali A. (2020). Vitamin D Exerts Neuroprotection via SIRT1/nrf-2/NF-kB Signaling Pathways against D-Galactose-Induced Memory Impairment in Adult Mice. Neurochem. Int. 142 (2021), 104893. 10.1016/j.neuint.2020.104893 33159979

[B4] Carabotti M. Scirocco A. Maselli M. A. Severi C. (2015). The Gut-Brain axis: Interactions between Enteric Microbiota, central and Enteric Nervous Systems. Ann. Gastroenterol. 28, 203–209. 25830558 PMC4367209

[B5] Gräff J. Woldemichael B. T. Berchtold D. Dewarrat G. Mansuy I. M. (2012). Dynamic Histone marks in the hippocampus and Cortex Facilitate Memory Consolidation. Nat. Commun. 3, 991. 10.1038/ncomms1997 22871810

[B6] He C. Huang Z. S. Yu C. C. Wang X. S. Tao J. Wu M. (2021). Preventive Electroacupuncture Ameliorates D-Galactose-Induced Alzheimer's Disease-like Inflammation and Memory Deficits, Probably via Modulating Themicrobiota-Gut-Brain axis, Iran. J. Basic Med. Sci. 24 (3), 341–348. 10.22038/ijbms.2021.49147.11256 PMC8087854 33995945

[B7] Higarza S. G. Arboleya S. Arias J. L. Gueimonde M. Arias N. (2021). Akkermansia Muciniphila and Environmental Enrichment Reverse Cognitive Impairment Associated with High-Fat High-Cholesterol Consumption in Rats. Gut Microbes 13 (1), 1–20. 10.1080/19490976.2021.1880240 PMC7946069 33678110

[B8] Hu Y. Li C. Shen W. (2014). Gastrodin Alleviates Memory Deficits and Reduces Neuropathology in a Mouse Model of Alzheimer's Disease. Neuropathology 34, 370–377. 10.1111/neup.12115 24661139

[B9] Iraji A. Khoshneviszadeh M. Firuzi O. Khoshneviszadeh M. Edraki N. (2020). Novel Small Molecule Therapeutic Agents for Alzheimer Disease: Focusing on BACE1 and Multi-Target Directed Ligands. Bioorg. Chem. 97, 103649. 10.1016/j.bioorg.2020.103649 32101780

[B10] Jamar G. Ribeiro D. A. Pisani L. P. (2021). High-fat or High-Sugar Diets as Trigger Inflammation in the Microbiota-Gut-Brain axis. Crit. Rev. Food Sci. Nutr. 61 (5), 836–854. 10.1080/10408398.2020.1747046 32267169

[B11] Jang J. H. Son Y. Kang S. S. Bae C. S. Kim J. C. Kim S. H. (2015). Neuropharmacological Potential of Gastrodia Elata Blume and its Components. Evid. Based Complement. Alternat Med. 2015, 309261. 10.1155/2015/309261 26543487 PMC4620291

[B12] Jeong J.-H. Koo J.-H. Yook J. S. Cho J.-Y. Kang E.-B. (2021). Neuroprotective Benefits of Exercise and Mitoq on Memory Function, Mitochondrial Dynamics, Oxidative Stress, and Neuroinflammation in D-Galactose-Induced Aging Rats. Brain Sci. 11 (2), 164. 10.3390/brainsci11020164 33514020 PMC7910851

[B13] Jiang Y. Li K. Li X. Xu L. Yang Z. (2021). Sodium Butyrate Ameliorates the Impairment of Synaptic Plasticity by Inhibiting the Neuroinflammation in 5XFAD Mice. Chem. Biol. Interact 341, 109452. 10.1016/j.cbi.2021.109452 33785315

[B14] Kennedy P. J. Cryan J. F. Dinan T. G. Clarke G. (2017). Kynurenine Pathway Metabolism and the Microbiota-Gut-Brain axis. Neuropharmacology 112, 399–412. 10.1016/j.neuropharm.2016.07.002 27392632

[B15] Kowalski K. Mulak A. (2019). Brain-Gut-Microbiota Axis in Alzheimer's Disease. J. Neurogastroenterol Motil. 25 (1), 48–60. 10.5056/jnm18087 30646475 PMC6326209

[B16] Kumar A. (2018). Editorial: Neuroinflammation and Cognition. Front. Aging Neurosci. 10, 413. 10.3389/fnagi.2018.00413 30618719 PMC6297877

[B17] Lee J.-G. Moon S.-O. Kim S.-Y. Yang E.-J. Min J.-S. An J.-H. (2015). Rapid HPLC Determination of Gastrodin in Gastrodiae Rhizoma. J. Korean Soc. Appl. Biol. Chem. 58, 409–413. 10.1007/s13765-015-0058-2

[B18] Li J. Gao L. Sun K. Xiao D. Li W. Xiang L. (2016). Benzoate Fraction from Gentiana Rigescens Franch Alleviates Scopolamine-Induced Impaired Memory in Mice Model *In Vivo* . J. Ethnopharmacol. 193, 107–116. 10.1016/j.jep.2016.08.001 27492328

[B19] Lin Y. Kotakeyama Y. Li J. Pan Y. Matsuura A. Ohya Y. (2019). Cucurbitacin B Exerts Antiaging Effects in Yeast by Regulating Autophagy and Oxidative Stress. Oxid. Med. Cel. Longev. 2019, 4517091. 10.1155/2019/4517091 PMC6589324 31281576

[B20] Liu J. Sun J. Wang F. Yu X. Ling Z. Li H. (2015). Neuroprotective Effects of Clostridium Butyricum against Vascular Dementia in Mice via Metabolic Butyrate. Biomed. Res. Int. 2015, 412946. 10.1155/2015/412946 26523278 PMC4615854

[B21] Liu J. Zhang T. Wang Y. Si C. Wang X. Wang R. T. (2020). Baicalin Ameliorates Neuropathology in Repeated Cerebral Ischemia-Reperfusion Injury Model Mice by Remodeling the Gut Microbiota. Aging (Albany NY) 12 (4), 3791–3806. 10.18632/aging.102846 32084011 PMC7066900

[B22] Liu X. Wu C. Han D. Liu J. Liu H. Jiang Z. (2019). Partially Hydrolyzed Guar Gum Attenuates D-Galactose-Induced Oxidative Stress and Restores Gut Microbiota in Rats. Int. J. Mol. Sci. 20 (19), 4861. 10.3390/ijms20194861 PMC6801633 31574948

[B23] Liu Y. Gao J. Peng M. Meng H. Ma H. Cai P. (2018). A Review on central Nervous System Effects of Gastrodin. Front. Pharmacol. 9, 24. 10.3389/fphar.2018.00024 29456504 PMC5801292

[B24] Lubomski M. Xu X. Holmes A. J. Yang J. Y. H. Sue C. M. Davis R. L. (2021). The Impact of Device-Assisted Therapies on the Gut Microbiome in Parkinson's Disease. J. Neurol. 269, 780–795. 10.1007/s00415-021-10657-9 34128115

[B25] Marizzoni M. Cattaneo A. Mirabelli P. Festari C. Lopizzo N. Nicolosi V. (2020). Short-Chain Fatty Acids and Lipopolysaccharide as Mediators between Gut Dysbiosis and Amyloid Pathology in Alzheimer's Disease. J. Alzheimers Dis. 78 (2), 683–697. 10.3233/JAD-200306 33074224

[B26] Murros K. E. Huynh V. A. Takala T. M. Saris P. E. J. (2021). Desulfovibrio Bacteria Are Associated with Parkinson's Disease. Front. Cel. Infect. Microbiol. 11, 652617. 10.3389/fcimb.2021.652617 PMC8126658 34012926

[B27] Obrenovich M. Jaworski H. Tadimalla T. Mistry A. Sykes L. Perry G. (2020). The Role of the Microbiota-Gut-Brain axis and Antibiotics in ALS and Neurodegenerative Diseases. Microorganisms 8 (5), 784. 10.3390/microorganisms8050784 PMC7285349 32456229

[B28] Oskouei Z. Mehri S. Kalalinia F. Hosseinzadeh H. (2021). Evaluation of the Effect of Thymoquinone in D ‐galactose‐induced Memory Impairments in Rats: Role of MAPK , Oxidative Stress, and Neuroinflammation Pathways and Telomere Length. Phytotherapy Res. 35 (4), 2252–2266. 10.1002/ptr.6982 33325602

[B29] Ou Z. Deng L. Lu Z. Wu F. Liu W. Huang D. (2020). Protective Effects of Akkermansia Muciniphila on Cognitive Deficits and Amyloid Pathology in a Mouse Model of Alzheimer's Disease. Nutr. Diabetes 10 (1), 12. 10.1038/s41387-020-0115-8 32321934 PMC7176648

[B30] Preston A. R. Eichenbaum H. (2013). Interplay of hippocampus and Prefrontal Cortex in Memory. Curr. Biol. 23 (17), R764–R773. 10.1016/j.cub.2013.05.041 24028960 PMC3789138

[B31] Sadovnikova I. S. Gureev A. P. Ignatyeva D. A. Gryaznova M. V. Chernyshova E. V. Krutskikh E. P. (2021). Nrf2/ARE Activators Improve Memory in Aged Mice via Maintaining of Mitochondrial Quality Control of Brain and the Modulation of Gut Microbiome. Pharmaceuticals (Basel) 14 (7), 607. 10.3390/ph14070607 34201885 PMC8308546

[B32] Seregin S. S. Golovchenko N. Schaf B. Chen J. Pudlo N. A. Mitchell J. (2017). NLRP6 Protects Il10 −/− Mice from Colitis by Limiting Colonization of Akkermansia Muciniphila. Cell Rep. 19, 733–745. 10.1016/j.celrep.2017.03.080 28445725 PMC5528001

[B33] Sharma V. K. Singh T. G. Garg N. Dhiman S. Gupta S. Rahman M. H. (2021). Dysbiosis and Alzheimer's Disease: A Role for Chronic Stress? Biomolecules 11 (5), 678. 10.3390/biom11050678 33946488 PMC8147174

[B34] Silva D. F. Candeias E. Esteves A. R. Magalhães J. D. Ferreira I. L. Nunes-Costa D. (2020). Microbial BMAA Elicits Mitochondrial Dysfunction, Innate Immunity Activation, and Alzheimer's Disease Features in Cortical Neurons. J. Neuroinflammation 17 (1), 332–418. 10.1186/s12974-020-02004-y 33153477 PMC7643281

[B35] Tan L. Y. Yeo X. Y. Bae H. G. Lee D. P. S. Ho R. C. Kim J. E. (2021). Association of Gut Microbiome Dysbiosis with Neurodegeneration: Can Gut Microbe-Modifying Diet Prevent or Alleviate the Symptoms of Neurodegenerative Diseases? Life (Basel) 11 (7), 698. 10.3390/life11070698 34357070 PMC8305650

[B36] Tükel Ç. Nishimori J. H. Wilson R. P. Winter M. G. Keestra A. M. Van Putten J. P. M. (2010). Toll-like Receptors 1 and 2 Cooperatively Mediate Immune Responses to Curli, a Common Amyloid from Enterobacterial Biofilms. Cell. Microbiol. 12 (10), 1495–1505. 10.1111/j.1462-5822.2010.01485.x 20497180 PMC3869100

[B37] United Nations (2019). Department of Economic and Social Affairs, Population Division, World Population Ageing. Available at: https://www.un.org/en/development/desa/population/publications/pdf/ageing/WorldPopulationAgeing2019-Highlights.pdf .

[B38] Varatharaj A. Galea I. (2017). The Blood-Brain Barrier in Systemic Inflammation. Brain Behav. Immun. 60, 1–12. 10.1016/j.bbi.2016.03.010 26995317

[B39] Virgili J. Lebbadi M. Tremblay C. St-Amour I. Pierrisnard C. Faucher-Genest A. (2018). Characterization of a 3xTg-AD Mouse Model of Alzheimer's Disease with the Senescence Accelerated Mouse Prone 8 (SAMP8) Background. Synapse 72, e22025. 10.1002/syn.22025 29341269

[B40] Wu L. Han Y. Zheng Z. Zhu S. Chen J. Yao Y. (2021). Obeticholic Acid Inhibits Anxiety via Alleviating Gut Microbiota-Mediated Microglia Accumulation in the Brain of High-Fat High-Sugar Diet Mice. Nutrients 13 (3), 940–1021. 10.3390/nu13030940 33803974 PMC7999854

[B41] Xiang L. Wu Q. Osada H. Yoshida M. Pan W. Qi J. (2020). Peanut Skin Extract Ameliorates the Symptoms of Type 2 Diabetes Mellitus in Mice by Alleviating Inflammation and Maintaining Gut Microbiota Homeostasis. Aging (Albany NY) 12 (14), 13991–14018. 10.18632/aging.103521 32699185 PMC7425515

[B42] Xiao Y. Dong J. Yin Z. Wu Q. Zhou Y. Zhou X. (2018). Procyanidin B2 Protects against D-Galactose-Induced Mimetic Aging in Mice: Metabolites and Microbiome Analysis. Food Chem. Toxicol. 119, 141–149. 10.1016/j.fct.2018.05.017 29751077

[B43] Zeng Y.-Q. Gu J.-H. Chen L. Zhang T.-T. Zhou X.-F. (2021). Gastrodin as a Multi-Target Protective Compound Reverses Learning Memory Deficits and AD-like Pathology in APP/PS1 Transgenic Mice. J. Funct. Foods 77, 104324. 10.1016/j.jff.2020.104324

[B44] Zhang D. Han J. Li Y. Yuan B. Zhou J. Cheong L. (2018). Tuna Oil Alleviates D-Galactose Induced Aging in Mice Accompanied by Modulating Gut Microbiota and Brain Protein Expression. J. Agric. Food Chem. 66, 5510–5520. 10.1021/acs.jafc.8b00446 29656644

[B45] Zhou Y. Wang Y. Quan M. Zhao H. Jia J. (2021). Gut Microbiota Changes and Their Correlation with Cognitive and Neuropsychiatric Symptoms in Alzheimer's Disease. Jad 81 (2), 583–595. 10.3233/jad-201497 33814442

