# Supplementary figures and images for: Gastrodin From Gastrodia elata Enhances Cognitive Function and Neuroprotection of AD Mice via the Regulation of Gut Microbiota Composition and Inhibition of Neuron Inflammation
Source: Front Pharmacol. 2022 Jun 2;13:814271. doi: 10.3389/fphar.2022.814271 (PMC9201506; doi:10.3389/fphar.2022.814271)

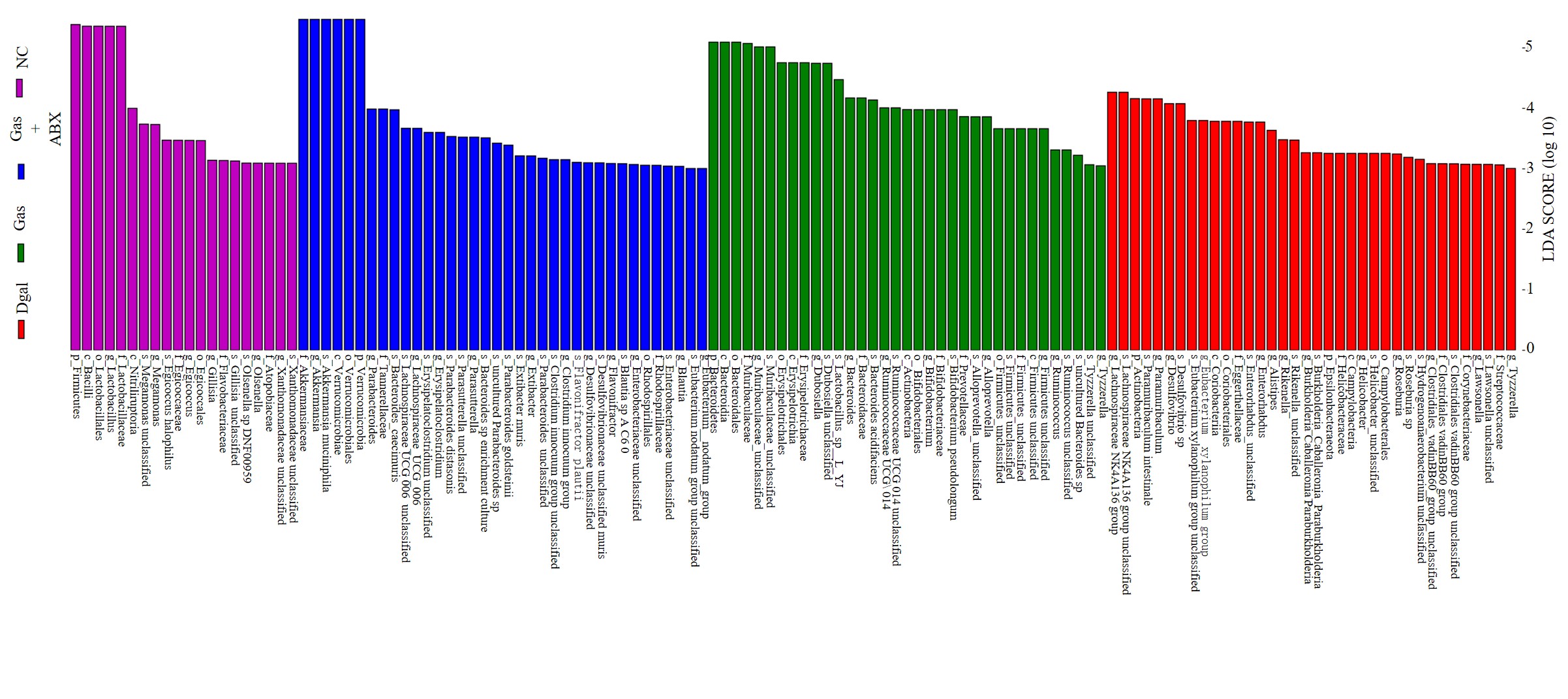

Supplement: Supplementary file 1 [file Image3.JPEG]

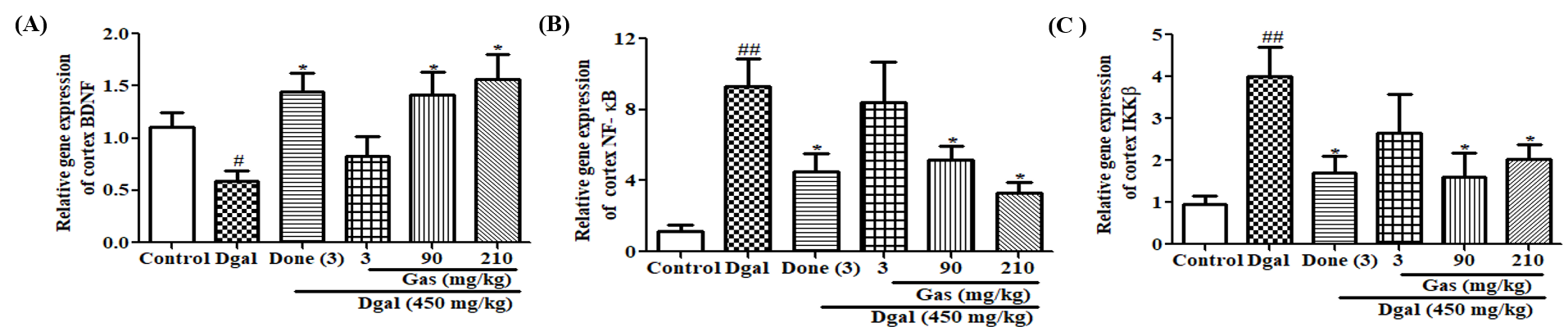

Supplement: Supplementary file 2 [file Image1.JPEG]

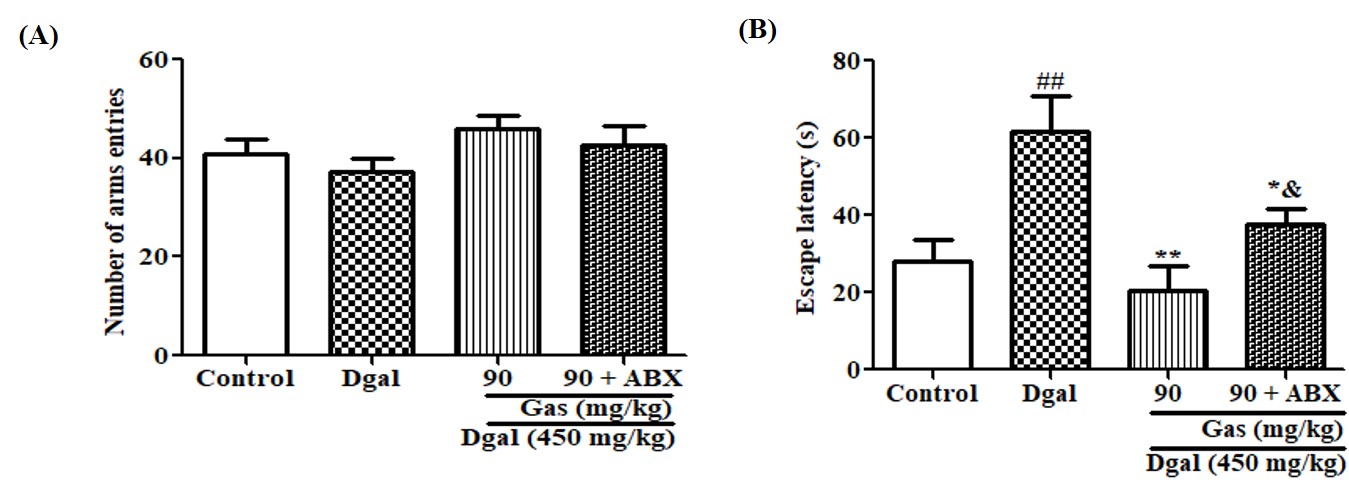

Supplement: Supplementary file 3 [file Image4.JPEG]

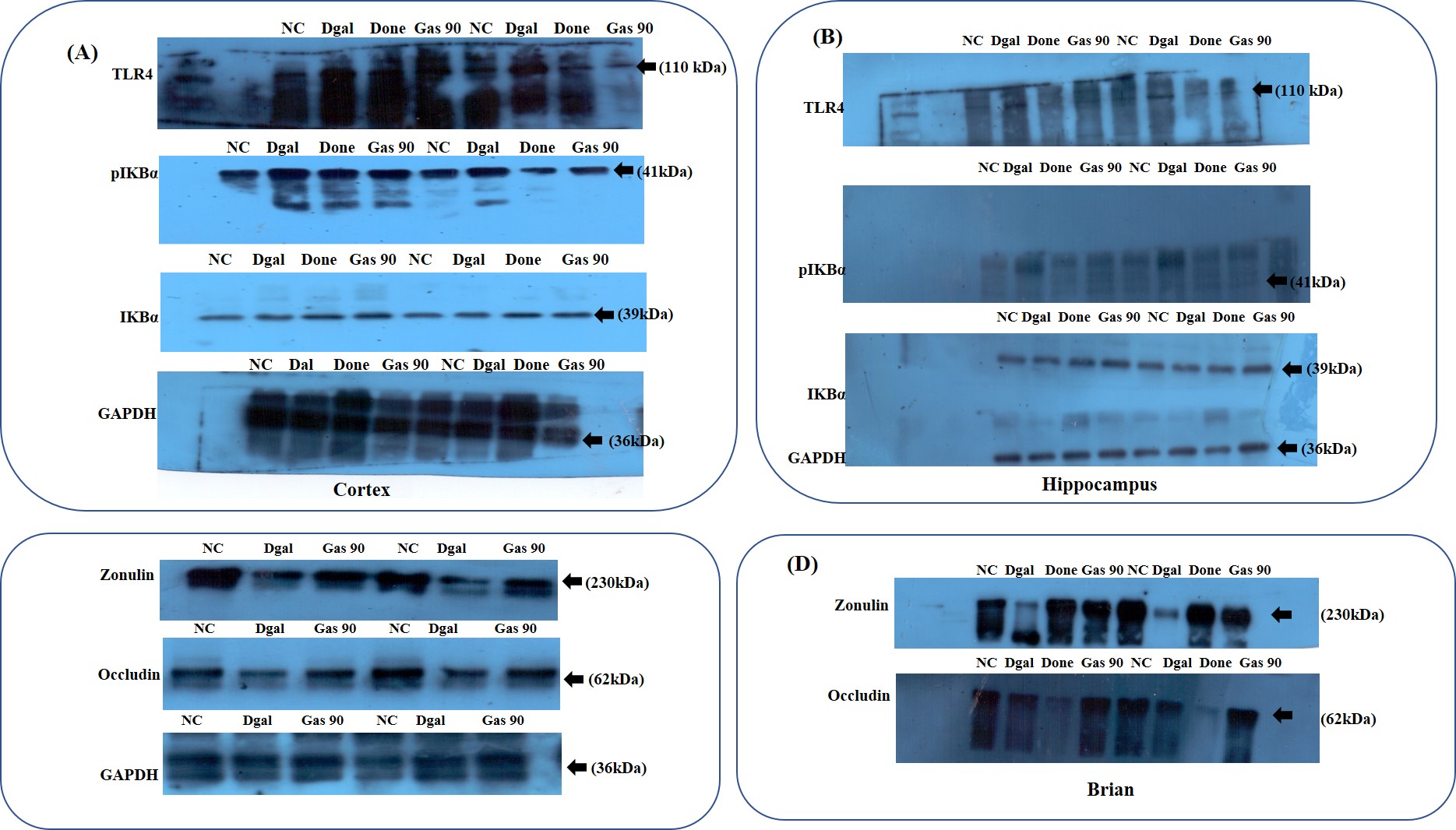

Supplement: Supplementary file 4 [file Image2.JPEG]

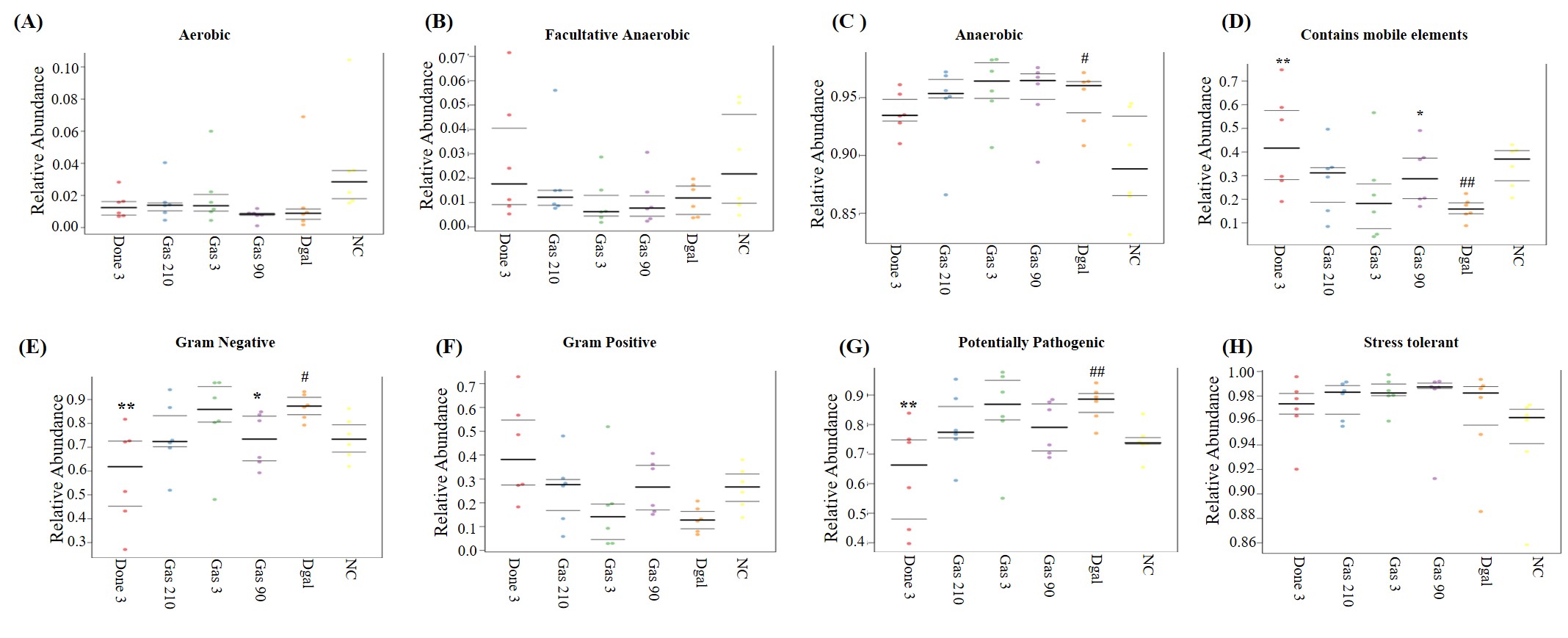

Supplement: Supplementary file 5 [file Image5.JPEG]

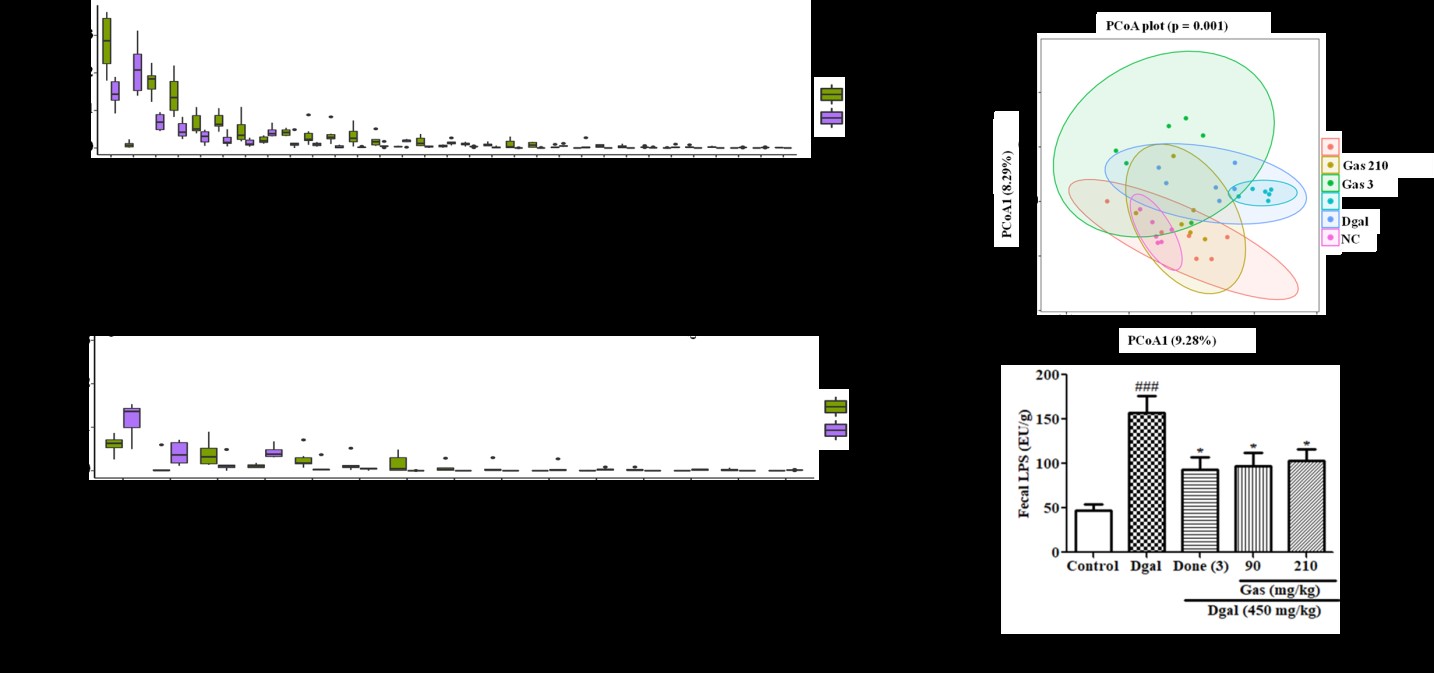

Supplement: Supplementary file 7 [file Image6.JPEG]
