# Supplementary material for: Gastrodin From Gastrodia elata Enhances Cognitive Function and Neuroprotection of AD Mice via the Regulation of Gut Microbiota Composition and Inhibition of Neuron Inflammation
Source: Front Pharmacol. 2022 Jun 2;13:814271. doi: 10.3389/fphar.2022.814271 (PMC9201506; doi:10.3389/fphar.2022.814271)
Supplement: Supplementary file 6 [file DataSheet1.docx]

**Supplementary information**

**Gastrodin from *Gastrodia elata* enhances cognitive function and neuroprotection of AD mice via the regulation of gut microbiota composition and inhibition of neuron inflammation**

Opeyemi Fasina^1^, Jianyu Wang^1^, Jianxia Mo^1^, Hiroyuki Osada^2^, Hiroshi Ohno^3^, Wensheng Pan^4^ Lan Xiang^1^* and Jianhua Qi^1^*

*^1^College of Pharmaceutical Science, Zhejiang University, 866 Yu Hang Road, Hangzhou, China*

*^2^* *Chemical Biology Research Group, RIKEN Center for Sustainable Resource Science, Wako-shi, Saitama 351-0198, Japan*

*^3^ Laboratory for Intestinal Ecosystem, RIKEN Center for Intestinal Ecosystem, Yokohama, 230- 0045, Japan*

*^4^* *Department of Gastroenterology, Zhejiang provincial people's hospital, People's Hospital of Hangzhou Medical College, Hangzhou 310014, Zhejiang Province, China*

*Correspondence should be addressed to Lan Xiang, [lxiang@zju.edu.cn](mailto:lxiang@zju.edu.cn); Jianhua Qi; qijianhua@zju.edu.cn

**Supplementary Table 1. Primers sequences used for RT-PCR analysis in this study**

| Gene | Species | Sequences |
| --- | --- | --- |
| NF-κb | Mouse | sense: 5’-AGC ACA GAT ACC AAG AC-3’  anti-sense: 5’-TCA GCC TCA TAG CCA TC -3’ |
| IKKβ | Mouse | sense: 5’-CGA GCC GCC ATG ATG AAT CT-3’  anti-sense: 5’-GCA GTT TAT CTG ATG TGA TCC CA -3’ |
| BDNF | Mouse | sense: 5’- TTG TTT TGT GCC GTT TAC CA -3’  anti-sense: 5’- GGT AAG AGA GCC AGC CAC TG -3’ |
| 18S | Mouse | sense: 5’-TAA CCC GTT GAA CCC CAT T -3’  anti-sense: 5’-CCA TCC AAT CGG TAG TAG CG -3’ |


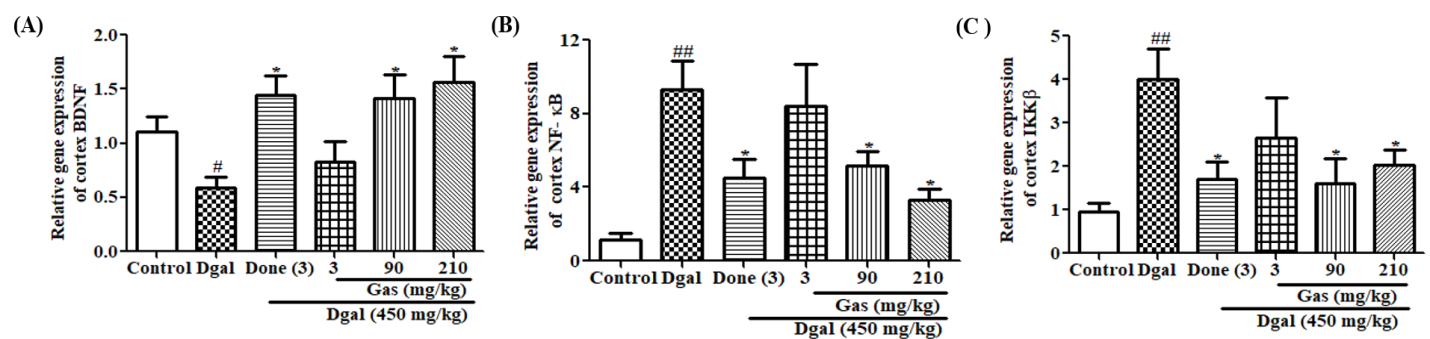


**Supplementary Figure 1**. **Effect of Gas on BDNF, NF-κb and IKKβ gene expression in cerebral cortex of AD mice.** Changes in BDNF (A), NF-κb (B) and IKKβ (C) genes expression in cerebral cortex of AD mice after administrating Gas. Each group has five sample and the data were presented as means ± SEM. ^#, ##^ indicate significant differences at *p* < 0.05 and *p* < 0.01 compared with the normal control; ^*^indicate significant difference at *p* < 0.05 compared with Dgal group.


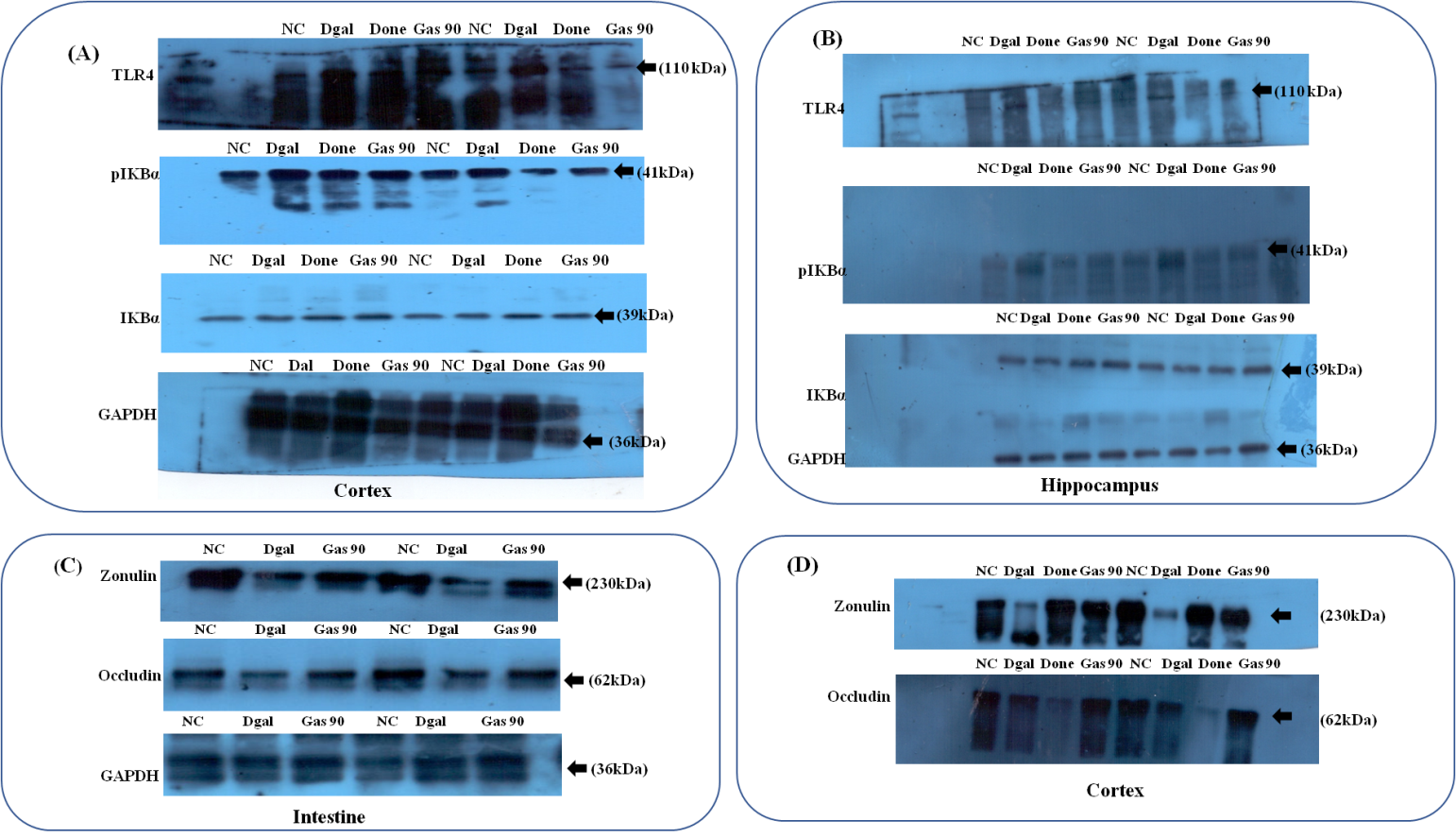


**Supplementary Figure 2. Original western blot analysis of Figure 5 and Figure 6.** Original western blot of TLR4, pIKBα, IKBα and GADPH of cortex (A) and hippocampus (B) in Figure 5. Original western blot of zonulin and occludin in intestine (C) and cortex (D) in Figure 6


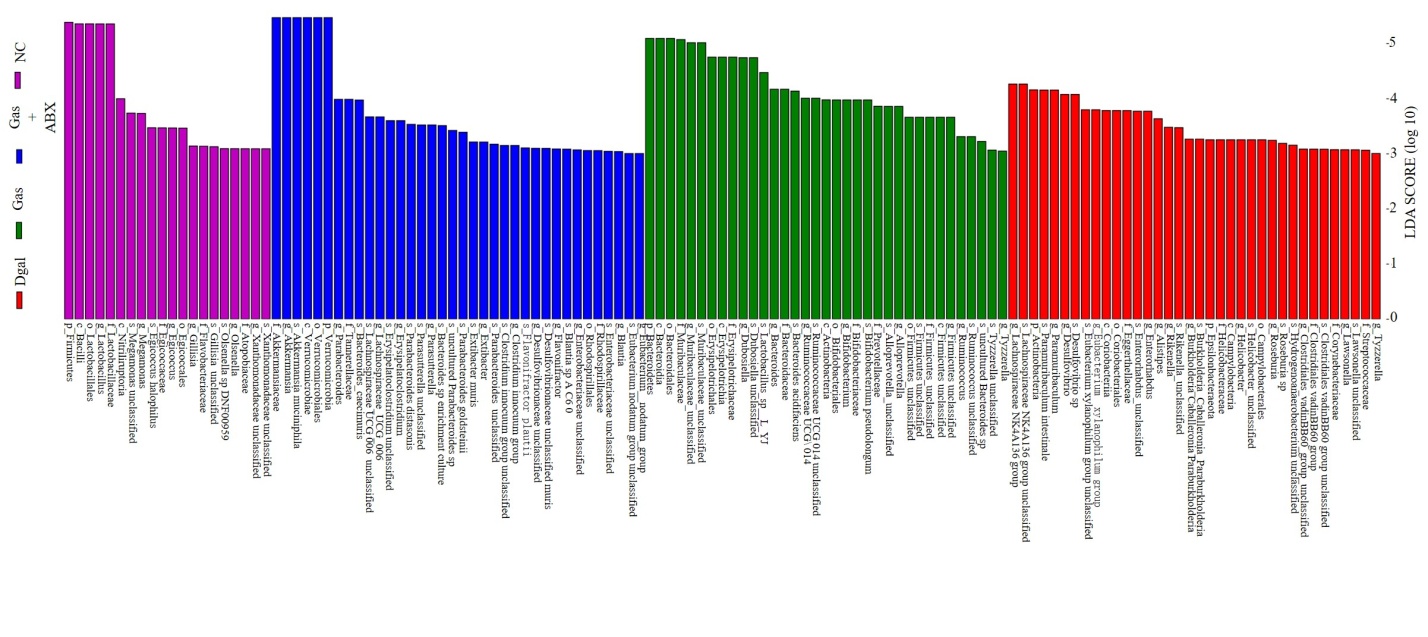


**Supplementary Figure 3**. **Effect of Gas on the composition of gut microbiota.** LEFSE analysis of gut microbiota with LDA scores greater than 3 as cut off point.


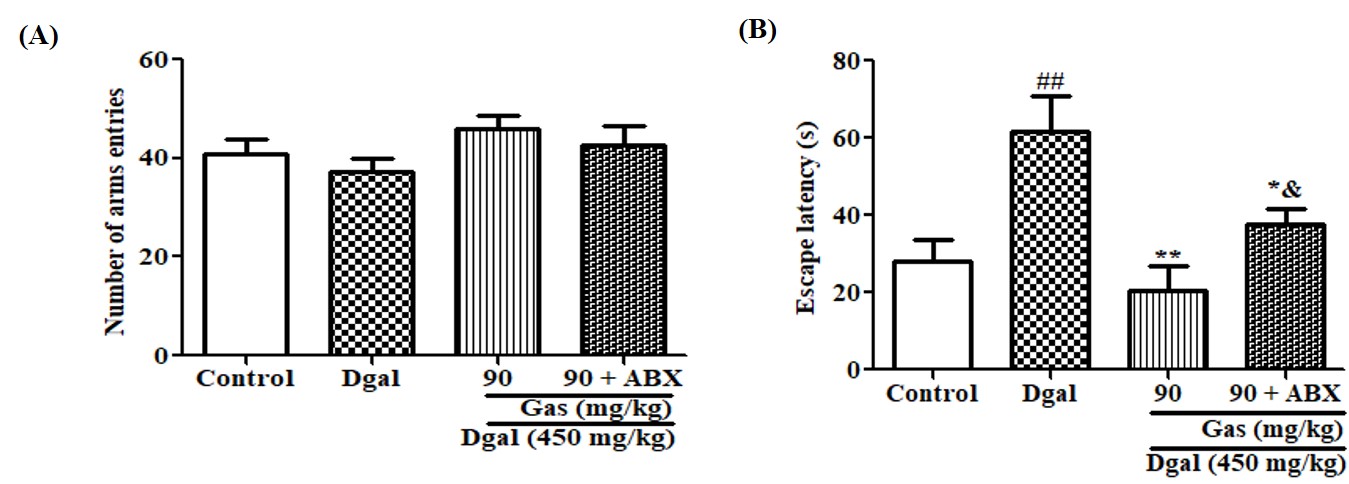


**Supplementary Figure 4**. **Effect of GAS on the gut microbiota depleted AD mice.** (A) Changes of the number of arms entries in Y maze test. (B) escape latency in test phase of water maze experiment. Each group has five mice and the data were presented as means ± SEM. ^##^ indicate significant differences at *p* < 0.01 compared with the normal control; ^* and **^ indicate significant difference at *p* < 0.05 and *p* < 0.01 compared with Dgal group. ^&^ represents significant difference at *p* < 0.05 compared with Gas group.


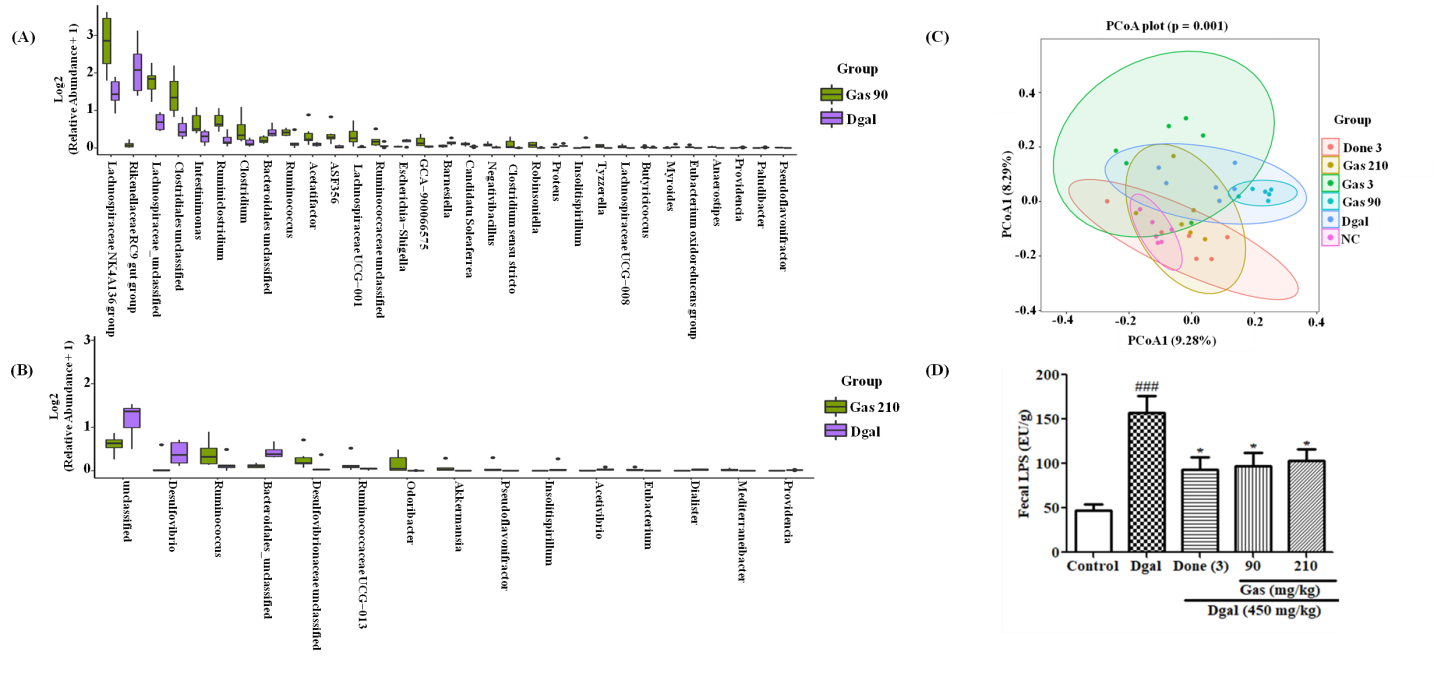


**Supplementary Figure 5**. **Effect of gastrodin on gut microbiota composition, and fecal LPS in AD mice.** Gut microbiota at genus level that significantly increased or decreased in Gas 90 mg/kg group (A) and Gas 210 mg/kg group (B) as compared with Dgal group. (C) PCoA analysis of gut microbiota in all experimental groups. (D) The changes in the fecal LPS of mice after giving Gas and Done, respectively. The fecal samples of each group are six or five, and the data are presented as means ± SEM. ^###^ indicate significant differences at *p* < 0.001 compared with the normal control; ^*^ indicate significant difference at *p* < 0.05 compared with Dgal group.


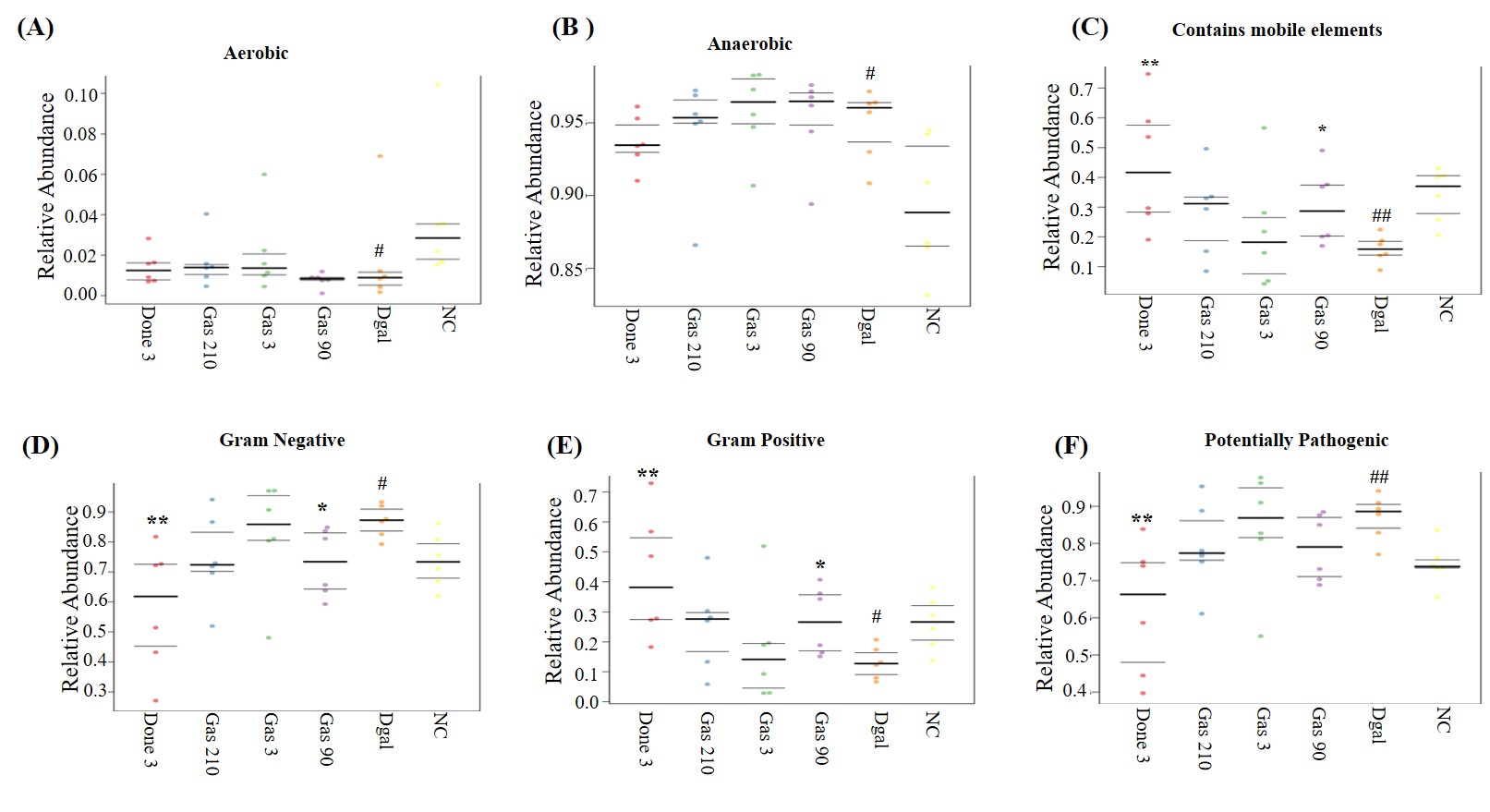


**Supplementary Figure 6**. **Effect of Gas on gut microbiota phenotypic predictions.** Graphical representation of gut microbiota predicted phenotypes such as (A) Aerobic, (B) Anaerobic, (C) Contains mobile elements, (D) Gram Negative, (E) Gram Positive, (F) Potentially Pathogenic in all experimental groups of AD mice.


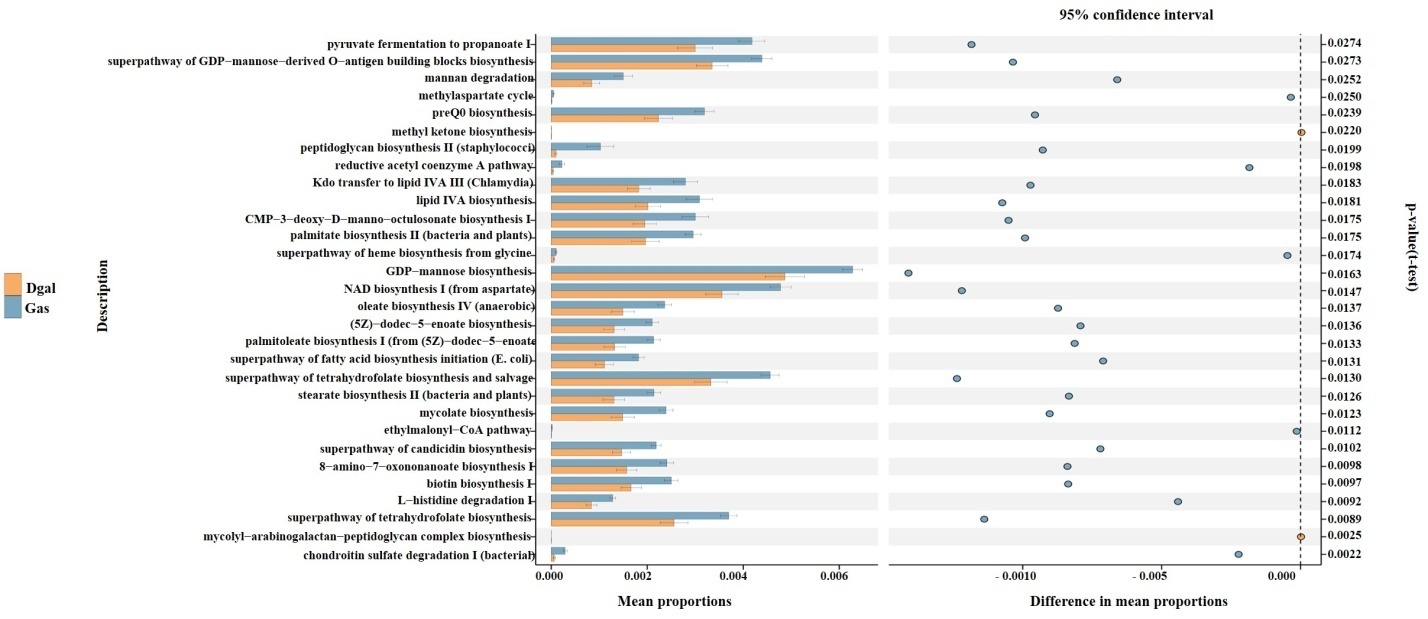


**Supplementary Figure 7**. **Effect of gastrodin on pathways.** Bar chart of species profiling of pathways with PICRUST analysis. Bar height shows mean proportion.
